# Nuclear ubiquitin-conjugating enzyme TrUbc4 and F-box protein TrFwd1-mediated modification of Cre1 in *Trichoderma reesei* establishes a regulatory mechanism for carbon catabolite repression

**DOI:** 10.1371/journal.pgen.1012216

**Published:** 2026-06-26

**Authors:** Gen Xu, Yanli Cao, Yuxiao Xia, Shanshan Jiang, Weixin Zhang, Xiangfeng Meng, Weifeng Liu

**Affiliations:** 1 State Key Laboratory of Microbial Technology, Microbial Technology Institute, Shandong University, Qingdao, People’s Republic of China; 2 School of Basic Medical Sciences, Jiangxi Medical College, Nanchang University, Nanchang, People’s Republic of China; Consejo Superior de Investigaciones Cientificas, SPAIN

## Abstract

Carbon catabolite repression (CCR) mediated by the transcriptional repressor Cre1 represents a major mechanism ensuring the energy-efficient cellulase production in the model cellulolytic fungus *Trichoderma reesei*. However, largely unknown is the regulatory pathway governing CCR. In this study, we identified a nuclear ubiquitination system targeting Cre1 to facilitate the induced cellulase gene expression. Either repression of *Trubc4* encoding an E2 (ubiquitin-conjugating enzyme) or deletion of *Trfwd1* encoding an F-box protein significantly compromised the induced cellulase biosynthesis. However, combinatorial repression of *cre1* suppressed the phenotypic defects resultant from mutations of *Trubc4 or Trfwd1*. Further analyses demonstrated that TrUbc4 and TrFwd1 collaboratively mediated the ubiquitination of Cre1. Impaired ubiquitination of Cre1 at K361 resulted in its enhanced binding to cellulase gene promoters even under cellulose inducing conditions. This persistent Cre1 binding in turn competitively excluded the functional promoter occupancy of the transcriptional activator Xyr1 required for full cellulase gene expression. These results thus support that Cre1 ubiquitination constitutes a primary mechanism to relieve CCR to ensure the efficient cellulase induction. The present work also highlights the importance of protein ubiquitination for control of carbohydrate utilization and biotechnologically relevant enzyme production in industrial filamentous fungi including *Trichoderma reesei.*

## Author summary

The efficient production of cellulolytic enzymes in industrial *Trichoderma reesei* is central to biomass conversion. This process, however, is tightly controlled by a regulatory mechanism named carbon catabolite repression (CCR), which ensures the cell to prioritize the utilization of readily metabolizable carbon sources like glucose. This is mainly achieved with a molecular switch involving a transcriptional repressor Cre1 by preventing cellulase gene expression in the presence of glucose even with appropriate environmental nutrient cues. A fundamental question therefore is how Cre1’s repression is relieved upon the depletion of the repressing carbon source to allow the cell ready to initiate the induced cellulase biosynthesis. Our study uncovered a nuclear-localized E2 enzyme TrUbc4 and an F-box protein TrFwd1, which participates in modulating Cre1’s DNA-binding capability to alleviate CCR and facilitating the transcription activator Xyr1-mediated cellulase gene expression. We also identified K361 as a critical ubiquitination site in Cre1 and revealed that this post-translational modification is necessary for its functional inactivation without an absolute prerequisite for its degradation. This work contributes to our understanding of the sophistication and diversity of post-translational control of eukaryotic gene transcription.

## Introduction

Posttranslational modifications (PTMs) play important roles in shaping protein functions, thereby participating in essential cellular processes as diverse as DNA repair, replication, gene transcription and cell differentiation [1,2]. One of such well-studied PTMs is protein ubiquitination in eukaryotic cells, a process that modifies target proteins by the covalent attachment of one or more small protein ubiquitin (Ub) [3,4]. Specific ubiquitination of the various substrates begins with E1 (ubiquitin-activating enzyme) that uses ATP to form a thioester bond between the carboxyl-terminus of ubiquitin and a catalytic cysteine residue in the active site. The E1 then engages an E2 (ubiquitin-conjugating enzyme) and transfers the activated ubiquitin to a cysteine residue in the E2 active site. Ub-charged E2 subsequently selectively interacts with an E3 (ubiquitin-protein ligase) that recruits and binds specific substrate proteins [5–7].

While E2 acts as a bridge to transfer the activated Ub to a target protein, it is E3 that ultimately determines the exquisite selectivity of ubiquitylation via its direct interaction with protein substrates. It is therefore not surprising to find that there normally exist a larger number of E3s compared to E1 and E2 [8–10]. E3 ligases generally fall into two main classes based on mechanism and domain architecture, single-subunit E3s (e.g., HECT, RING types) and multi-subunit E3s (e.g., APC/C, SCF complex) [11,12]. While HECT (homology to E6AP C-terminus) type E3s form a catalytic thioester intermediate with ubiquitin before it is transferred to protein substrates [13], RING (really interesting new gene) type E3s scaffold the direct ubiquitin transfer from E2 to target proteins [14]. Among the multi-subunit E3s, Cullin-RING Ligases (CRLs) constitute the largest superfamily of ubiquitin ligases that are formed around a cullin scaffold. The best-characterized member is the Cul1-RING E3 ligase, commonly known as the SCF complex with three invariable core components including Rbx1 (Ring-box protein 1) that recruits the E2 enzyme, Cul1 (Cullin 1) acting as the scaffold, and Skp1 (S-phase kinase-associated protein 1) that serves as an adaptor bridging the core SCF complex with a variable F-box protein (FBP) [12,15–17]. While several E2s can work with a same E3, the converse is true as well that a given E2 can interact with multiple E3s to achieve the high plasticity and complexity of ubiquitin-mediated regulation. The disproportionate number of E2s versus E3s together with the hierarchical nature of the ubiquitination reaction cascade, therefore also pose a formidable challenge in dissecting specific E2-E3 as well as E3-substrate interactions [18–20].

Among the various cellular processes involving protein ubiquitylation-mediated regulation, it is becoming increasingly clear that protein modification with Ub plays a significant part in regulating transcription [21]. While monoubiquitylation has been reported to promote the nuclear localization of a transcription factor to activate the transcription of downstream target genes [22], polyubiquitination-mediated proteolysis of transcriptional repressors or activators via 26S proteasome has been found not only to restrict but also in some cases to aid their function [23,24]. Specifically, ubiquitin-proteasome system (UPS) has been found to play important roles in promoting the transcriptional activity of *Saccharomyces cerevisiae* Gal4 in an “activation by destruction” manner although the exact mechanism remains elusive [25–27]. On the other hand, galactose-induced protein degradation of the repressor Mig2 by an SCF E3 ubiquitin ligase SCF^Das1^ has been also shown to be required for the efficient galactose induction of the *gal1* gene [28]. Interestingly, a recent study in *Penicillium oxalicum* revealed that deletion of an F-box protein leads to the accumulation of the transcriptional repressor Ace1, which however cannot bind its target genes, ultimately resulting in an elevated xylanase gene expression [29]. These results thus highlight the versatile roles of protein ubiquitination in regulating gene transcription ubiquitination.

The filamentous fungus *T. reesei* represents the primary industrial workhorse for cellulase production, which is capable of efficiently depolymerizing insoluble cellulose into fermentable sugars via synergistic catalysis, enabling sustainable production of second-generation biofuels and bio-based chemicals [30,31]. In *T. reesei*, cellulase gene expression is governed by a sophisticated regulatory network that enables their exquisite adaptation to complex environmental cues to ensure the energy-efficient production of cellulases and hemicellulases [32]. Among others, carbon catabolite repression (CCR) constitutes a major regulatory aspect that ensures the stringent control of the induced cellulase biosynthesis [33,34]. CCR has been also demonstrated as a highly conserved regulatory mechanism to repress production of the respective enzymes required for the utilization of alternative carbon sources in the presence of rapidly metabolizable sugars such as glucose in other fungi [35–37]. For the various transcription factors that have been thus far identified to participate in the stringent regulation of the expression of cellulase genes in *T. reesei* [38], Xyr1 (xylanase regulator 1) has been established as the key transcriptional activator for almost all cellulase and hemicellulase genes [39,40]. However, relatively little is known about the regulatory mechanism controlling CCR.

Fungal CCR is best characterized in the budding yeast *S. cerevisiae*, which has been found to be directly regulated by a Cys_2_His_2_-type transcription factor Mig1 [41]. Similarly, a homologous factor CreA/Cre1 has been found to be responsible for controlling CCR in filamentous fungi [42]. In *Aspergillus nidulans*, while CreA has been well established to mediate glucose repression of the ethanol regulon genes through competition with the AlcR-specific transactivator [43], it has been also proposed to participate in the repression of the proline utilization gene cluster resultant from the presence of both repressing nitrogen and carbon sources [44]. Although several other factors that are involved in post-translational modifications of Mig1 have been identified as CCR regulating factors, the underlying mechanisms by which PTMs regulate CCR in filamentous fungi in response to carbon signals remain poorly understood. Moreover, a shuttle between the nucleus and the cytoplasm induced by a change in glucose concentration has been observed for both Mig1 and CreA [45]. Notably, whereas phosphorylation and dephosphorylation of Mig1 effects its subcellular change [41], an analogous mechanism has not been revealed for CreA [46–48]. Apart from CreA, three other factors (CreB, CreC and CreD) have been implicated in regulating CCR in filamentous fungi. The observations that CreB and CreC form a deubiquitinating enzyme complex [49–51], and that CreD is an arrestin-like protein which may serve as an adaptor of ubiquitin ligase, have led to a hypothesis that UPS-mediated modification of CreA protein is involved in filamentous fungal CCR regulation [52]. Although genetic analyses indeed support an antagonistic balance between CreD and the CreB-CreC complex, definitive evidence verifying their involvement in CreA ubiquitination remains elusive. Nonetheless, studies in *A. nidulans* have implicated an F-box proteins Fbx23 in regulating CreA-mediated carbon CCR although direct evidence that CreA was indeed the target of an SCF complex and the exact role of its ubiquitination was still lacking [53].

This study aims to systematically elucidate the critical role of protein ubiquitination in modulating CCR and the induced cellulase gene expression in *T.* *reesei*. We identified a nucleus localized E2 enzyme, TrUbc4, and a pairing F-box protein, TrFwd1, which together constitute a unique nuclear ubiquitination cascade. We demonstrated that the indicated SCF ubiquitin ligase complex mediated the ubiquitination of the CCR repressor Cre1, with the K361 residue being identified as a critical ubiquitination site. This modification prompted Cre1 dissociation from cellulase gene promoters on cellulose induction, thereby relieving its interference with the binding of the master activator Xyr1 to enable cellulase gene transcription. This work reveals a unique PTM-mediated molecular switch in the fungal CCR regulatory network and provides novel targets for further investigations in alternative carbon utilization in biotechnologically relevant fungi.

## Results

### The ubiquitin-conjugating enzyme TrUbc4 contributes to cellulase gene expression

The yeast one-hybrid screen was performed using a cDNA expression library prepared from *T. reesei* under cellulase-inducing conditions [54]. Since cellobiohydrolase I (CBH1) is the main enzymatic component that is highly induced upon induction, a *cbh1* promoter region (–474 to –838 bp) was applied to drive the expression of an Aureobasidin A (AbA) resistance gene *AUR1-C* for screening for additional transcriptional regulators. Six out of approximately 10^5^ transformants were obtained from the selective plates containing AbA. One clone contained a cDNA sequence encoding a 147‑amino‑acid polypeptide with an ATG start codon and was revealed to correspond to a ubiquitin-conjugating enzyme (E2) belonging to the Ubc4/5 subfamily (hereafter named TrUbc4, GenBank: XP_006968852.1, Tr123773) (S1A Fig). The E2 activity of TrUbc4 was determined by an *in vitro* Ub conjugation assay, wherein the formation of the TrUbc4-Ub complex as well as the ubiquitin dimer were observed when ATP, E1, Ub, and GST-TrUbc4 were simultaneously available (S1B Fig).

**Fig 1:**
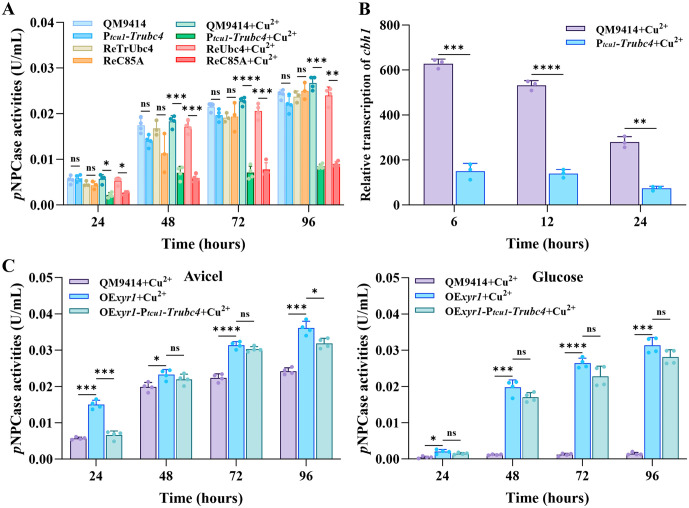
Repression of *Trubc4* compromised the induced cellulase gene expression. **(A)** Extracellular *p*NPC hydrolytic activity of P_*tcu1*_*-Trubc4,* as well as P_*tcu1*_*-Trubc4* complemented with the TrUbc4 (ReTrUbc4) or C85A mutant (ReC85A) at the *pyr4* locus under the control of the *tef1* promoter, cultured on 1% Avicel with or without copper. **(B)** Quantitative RT-PCR analyses of the transcription of the *cbh1* gene in the P_*tcu1*_*-Trubc4* strain cultured on 1% Avicel with adding copper to repress *Trubc4*. **(C)** Extracellular *p*NPC hydrolytic activity of the P_*tcu1*_*-Trubc4* strain overexpressing *xyr1* by the *cdna1* promoter (OE*xyr1*-P_*tcu1*_-*Trubc4*) cultured on 1% Avicel (left) or 1% glucose (right) with added copper to repress *Trubc4*. Data are represented as mean ± SD. ^ns^P > 0.05, ^*^P < 0.05, ^**^P < 0.01, ^***^P < 0.001, ^****^P < 0.0001.

Repeated efforts to obtain the *Trubc4* null mutant (Δ*Trubc4*) to see whether *Trubc4* affects the induced cellulase gene expression failed, implicating that *Trubc4* is likely an essential gene for fungal growth. We therefore applied an alternative strategy by replacing the *Trubc4* promoter with a copper-responsive promoter *tcu1*, which represses gene expression in the presence of copper while leading to constitutively high expression without addition of copper [40]. While growth of the resultant P_*tcu1*_*-Trubc4* strain showed slightly reduced mycelium diameter and density on glucose plate without copper, hardly any growth difference was observed in liquid glucose media regardless of *Trubc4* repression or overexpression compared to the control strain QM9414 (S2A and S2B Fig). Analyses of the extracellular (hemi)cellulase activities revealed that repression of *Trubc4* exhibited a pronounced reduction (approximately 65%) in *p*NPC (p-nitrophenyl-β-D-cellobioside), *p*NPG (p-nitrophenyl-β-D-glucopyranoside), CMC (carboxymethyl cellulose sodium salt) and xylan hydrolytic activities compared to QM9414 (Figs 1A and S2C and S2D). Further quantitative RT-PCR analyses revealed that *Trubc4* repression resulted in a significant down-regulation of main cellulase genes (*cbh1*, *eg1*, and *bgl1*) compared with the control strain QM9414 cultured on 1% Avicel (Figs 1B and S2E). Contrary to *Trubc4* repression, constitutively enhanced expression of *Trubc4* without copper led to hardly any effect on the extracellular cellulase activity of P_*tcu1*_*-Trubc4* compared to the control strain QM9414 (Figs 1A and S2C).

Since Blast analysis performed using the *S. cerevisiae* Ubc4 as a query retrieved a total of 19 other putative E2 enzymes in the *T. reesei* genome (S3A Fig), we individually knocked out these E2 genes in *T. reesei*. Unlike *Trubc4*, none except a Ubc8 mutant Δ*Tr65553* showed a remarkable decrease in the extracellular *p*NPC hydrolytic activity (S1 Table).

To verify the functional involvement of TrUbc4 as a ubiquitin-conjugating enzyme in regulating *T. reesei* cellulases, its evolutionarily conserved catalytic cysteine at 85 was substituted for alanine [55], and the C85A mutant was introduced back into P_*tcu1*_*-Trubc4* to generate the ReC85A strain. In contrast with the wild-type TrUbc4 complemented ReTrUbc4 strain, ReC85A failed to restore cellulase activity when copper was added to repress the endogenous *Trubc4* expression (Figs 1A and S2C), indicating that the E2 enzymatic activity of TrUbc4 is essential for its function in regulating cellulase gene expression*.* Notably, the extracellular *p*NPC hydrolytic activity of the ReC85A strain was comparable with that of the wild-type strain without addition of copper. This result therefore suggests that even with the expression of C85A mutant TrUbc4, the overexpressed wild-type TrUbc4 is sufficient to maintain the activty to ensure the induced cellulase gene expression (Fig 1A).

Since there exists a strict correlation between the transcription level of the critical transcriptional activator gene *xyr1* and cellulase genes [40,56], we enhanced *xyr1* expression to see whether it could rescue the induction defect due to the downregulation of *Trubc4*. The resulting strain with *xyr1* being expressed from a strong constitutive promoter *cdna1* (*Tr110879*) exhibited a full restoration of cellulase production under either glucose-repressing or Avicel-inducing conditions (Fig 1C). Altogether, these results suggest that TrUbc4 may function in a ubiquitination cascade to modulate Xyr1-mediated cellulase gene expression.

### TrUbc4 is a nuclear protein that requires a putative RING E3 complex to influence cellulase gene expression

**Fig 2:**
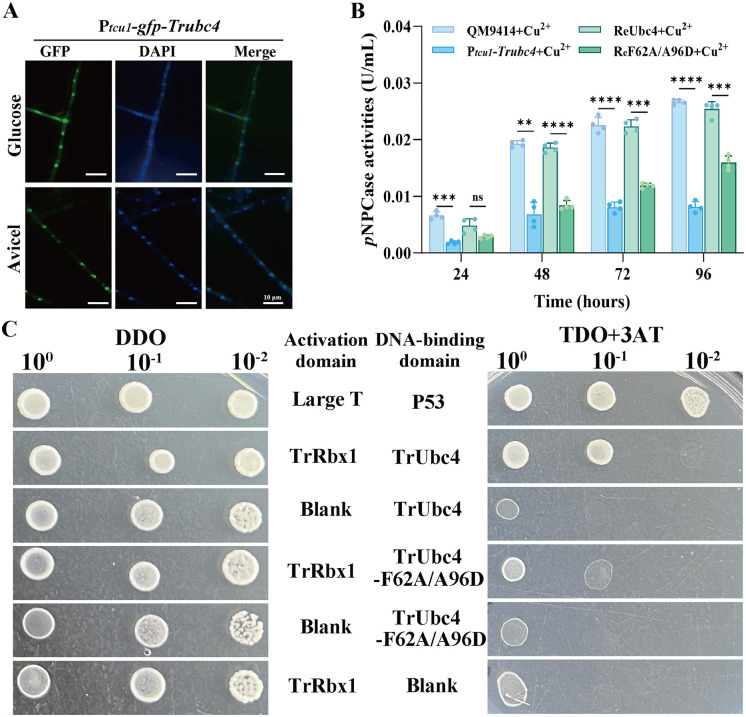
TrUbc4 localizes to the nucleus and interacts with TrRbx1. **(A)** Fluorescence microscopy analysis of the subcellular localization of the P_*tcu1*_*-gfp-Trubc4* strain cultured on glucose or Avicel conditions. **(B)** Extracellular *p*NPC activity of P_*tcu1*_-*Trubc4*, as well as P_*tcu1*_-*Trubc4* complemented with the TrUbc4 (ReUbc4) or F62A/A96D (ReF62A/A96D) mutant at the *pyr4* locus under the control of the *tef1* promoter, cultured on 1% Avicel with copper. **(C)** Protein interaction analysis of TrUbc4 or F62A/A96D mutant and TrRbx1 using yeast two-hybrid assay. Yeast cells harboring the indicated combinations of plasmids were plated on DDO lacking leucine and tryptophan or TDO lacking leucine, tryptophan, and histidine but containing 0.1 mM 3- amino-1,2,4-triazole (3AT). The P53/Large T combinations were used as positive. Tenfold serially diluted cell cultures were inoculated on each spot. Data are represented as mean ± SD. ^ns^P > 0.05, ^*^P < 0.05, ^**^P < 0.01, ^***^P < 0.001, ^****^P < 0.0001.

To determine the subcellular localization of TrUbc4 in *T. reesei*, green fluorescent protein (GFP) fused to the N-terminus of TrUbc4 was expressed under the control of the *tcu1* promoter. The fused GFP did not interfere with TrUbc4 function (S3B Fig), and GFP fluorescence was readily observed in the nucleus that well merged with signals stained by DAPI when the conidia were germinated on glucose (Fig 2A). This nuclear recruitment of TrUbc4 remained unchanged when the conidia were shifted from non-inducing (glucose) to inducing (Avicel) conditions.

Given that Phe62 and Ala96 in human UBC4 family have been shown to be critical for its specific interaction with RING E3 ligases [57,58], mutations of the corresponding amino acids in TrUbc4 were made and their effect on cellulase gene expression was evaluated. The introduction of the corresponding TrUbc4 mutant (F62A/A96D) into the P_*tcu1*_*-Trubc4* strain (ReF62A/A96D) hardly affected the growth (S2B Fig), but only partially rescued the phenotypic defect in cellulase production with *Trubc4* repression (Fig 2B). Moreover, a direct weak interaction was detected between TrUbc4 and TrRbx1, which is the only annotated *T. reesei* homolog (Tr121950) to the RING-finger subunit Rbx1 of the SCF complex (Fig 2C). The interaction was, however, almost abolished with the F62A/A96D double mutations in TrUbc4. Overall, these results strongly suggest that an active TrUbc4-RING E3 complex is involved in controlling the cellulase gene expression in *T. reesei*.

### Nuclear-localized F-box protein TrFwd1 participates in regulating cellulase gene expression

**Fig 3:**
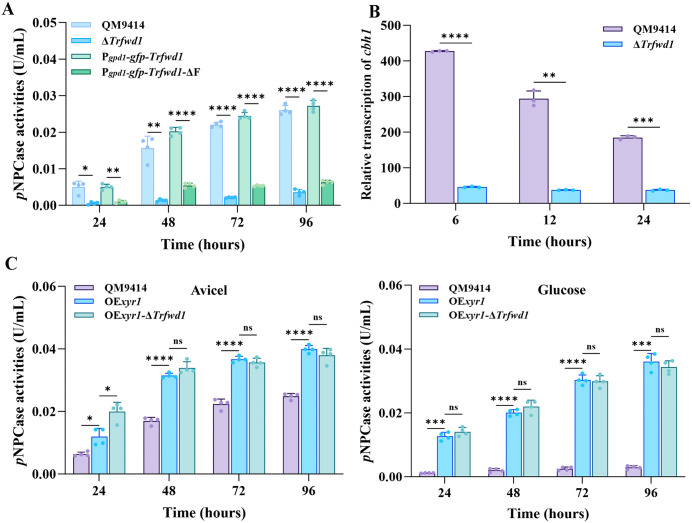
Deletion of *Trfwd1* compromised the induced cellulase gene expression which was resotred with *xyr1* overexpression. **(A)** Extracellular *p*NPC hydrolytic activity of the Δ*Trfwd1* strain as well as the deletion strain complemented with GFP-TrFwd1 (P_*gpd1*_*-gfp-Trfwd1*) or GFP-TrFwd1 without F-box domain (P_*gpd1*_*-gfp-Trfwd1*-ΔF) cultured on 1% Avicel. **(B)** Quantitative RT-PCR analyses of the *cbh1* gene in the *Trfwd1* knockout strain cultured on 1% Avicel. **(C)** Extracellular *p*NPC hydrolytic activity of the *Trfwd1* knockout strain with *xyr1* overexpression driven by *tcu1* promoter (OE*xyr1*-ΔTr*fwd1*) cultured on 1% Avicel (left) or 1% glucose (right). Data are represented as mean ± SD. ^ns^P > 0.05, ^*^P < 0.05, ^**^P < 0.01, ^***^P < 0.001, ^****^P < 0.0001.

Given the subcellular localization of TrUbc4, we focused on E3 ligases in *T. reesei* with putative nuclear localization signals predicted by NLStradamus (http://www.moseslab.csb.utoronto.ca/NLStradamus/) [59]. In silico analysis predicted a total of 103 E3 ligases in *T. reesei* genome, among which 44 were predicted to contain a putative nuclear localization signal (S2 Table). Homologous recombination-mediated gene knockout or copper-inducible promoter replacement strains were successfully constructed for 11 of the first 13 randomly selected E3 ligase genes. Phenotypic screening was then performed by determining the extracellular *p*NPC hydrolytic activity on 1% Avicel. This primary screening revealed that the absence of an F-box protein, TrFwd1 (GenBank accession: XP_006966975.1; gene ID: Tr79756), resulted in a phenotype similar to that observed under *Trubc4* repression (Fig 3A). TrFwd1 shares 48% identity with *N. crassa* Fwd1 and contains a conserved F-box domain at the N-terminus along with six WD40 repeats at the C-terminus (S4A Fig), supporting its classification as an integral component of a putative SCF E3 ubiquitin ligase complex. Phylogenetic analysis further indicated that TrFwd1 clusters with orthologs from various other filamentous fungi (S4B Fig).

While the hyphal growth of Δ*Trfwd1* was reduced regarding mycelium diameter and density compared to that of the control strain QM9414 on MM glucose plate, no apparent difference was observed in liquid glucose medium (S5A and S5B Fig). However, unlike repression of *Trubc4*, Δ*Trfwd1* produced hardly any conidia on malt extract medium (S5C Fig). Under cellulase-inducing or xylanase-inducing conditions, the extracellular *p*NPC, *p*NPG, CMC and xylan hydrolytic activities in Δ*Trfwd1* were decreased by approximately 80% relative to QM9414 (Figs 3A and S6A and S2D). Quantitative RT-PCR analyses further revealed a significant reduction in the transcription of key cellulase genes (*cbh1*, *eg1*, and *bgl1*) in Δ*Trfwd1* compared to that of the control strain QM9414 (Figs 3B and S6B). Similar to the repression of *Trubc4*, Xyr1 overexpression fully restored cellulase production under either glucose-repressing or Avicel-inducing conditions with *Trfwd1* deletion (Fig 3C). All the data therefore suggest that TrUbc4 couples with TrFwd1 to target a specific factor by ubiquitination to effect the key transcriptional activator-mediated cellulase gene expression.

**Fig 4:**
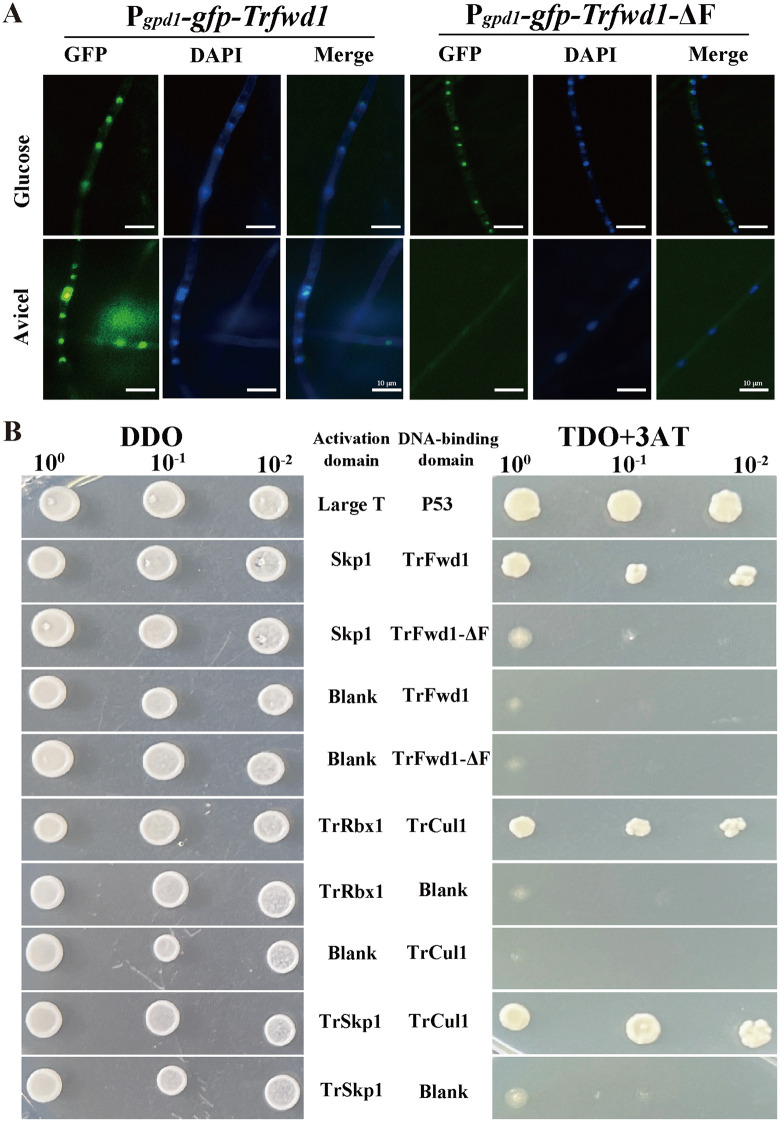
TrFwd1 localizes to the nucleus and is a component of a putative SCF complex. **(A)** Fluorescence microscopy analysis of the cellular localization of P_*gpd1*_*-gfp-Trfwd1* and P_*gpd1*_*-gfp-Trfwd1*-ΔF under glucose or Avicel conditions. **(B)** Protein interaction analysis of TrFwd1 and TrSkp1, TrRbx1 and TrCul1, TrCul1 and TrSkp1, using yeast two-hybrid assay. Yeast cells harboring the indicated combinations of plasmids were plated on DDO lacking leucine and tryptophan or TDO lacking leucine, tryptophan, and histidine but containing 0.5 mM 3AT. The P53/Large T combinations were used as positive. Tenfold serially diluted cell cultures were inoculated on each spot.

Analysis of the subcellular localization of TrFwd1 by expressing an N-terminal GFP-tagged TrFwd1 under the constitutive promoter *gpd1* (*Tr119735*) at *Trfwd1* locus in Δ*Trfwd1* revealed that GFP-TrFwd1 was fully functional in complementing Δ*Trfwd1* regarding defects in conidiation and cellulase production (Figs 3A, S5A, S5B, S5C, and S6A), and that the GFP fluorescence was predominantly localized to the nucleus under both glucose and Avicel conditions (Fig 4A). In contrast with GFP-TrFwd1, a corresponding strain expressing a TrFwd1 lacking the F-box domain (GFP-TrFwd1-ΔF) failed to restore conidiation and exhibited only a marginal recovery (~10%) in extracellular *p*NPC hydrolytic activity compared to Δ*Trfwd1* (Figs 3A, S5A, S5B, S5C, and S6A). Although GFP-TrFwd1-ΔF was similarly nuclear-localized, the detected fluorescence signal was much weaker than the full-length GFP-TrFwd1 (Fig 4A). Western blot analysis of the immunoprecipitation (IP) by GFP-Trap beads demonstrated that while GFP-TrFwd1 was readily detected, GFP-TrFwd1-ΔF was observed at markedly reduced levels (S6C Fig), indicating that the F-box domain is critical for TrFwd1 function, likely because it mediates the proper assembly of TrFwd1 into the SCF ubiquitin ligase complex, and its absence would destabilize the protein in its free-form.

Similar results were obtained for a TAP (tandem affinity purification) tagged TrFwd1 or TrFwd1-ΔF fusing with 5 × Myc-6 × His at its N-terminus to avoid any potential interference from GFP (S7A and S7B Fig). Furthermore, a yeast two-hybrid assay revealed that TrFwd1 specifically interacted with the adaptor TrSkp1 (Tr73823) of the SCF complex whereas this interaction was abolished upon deletion of the F-box domain (Fig 4B). Possibility that the instability of the mutant may account for this loss of interaction can not be excluded. Collectively, these results indicate that the F-box domain is critical for both the stability and normal functions of TrFwd1.

To further understand the regulatory influence of TrFwd1 on the induced cellulase biosynthesis, protein interaction studies using a cross-linking-based IP with the Myc-His-tagged TrFwd1 were performed followed by SDS-PAGE and silver staining. While DSP (dithiobis/succinimidylpropionate) crosslinking resulted in protein bands to be predominantly accumulated at the top of the gel, indicating the formation of high-molecular-weight complexes, decrosslinking resulted in the appearance of multiple distinct bands (S7C Fig). Mass spectrometry analysis of the crosslinked protein bands identified TrFwd1 as the most abundant protein in the complex compared to the control immunoprecipitation. Additionally, core SCF components including TrCul1 (Tr55706) and TrSkp1 as well as multiple subunits of the COP9 signalosome such as the catalytic subunit TrCsn5 (Tr62003) that are known to modulate the SCF complex activity, were also detected in the TrFwd1-tagged strain (S3 Table). Further yeast two-hybrid assays confirmed that TrCul1 directly interacted with both TrSkp1 and TrRbx1, supporting the presence of an integral SCF complex harboring TrFwd1 (Fig 4B).

To assess the functional involvement of the identified key subunits of the putative SCF complex in *T. reesei* cellulase gene expression, we constructed the promoter replacement strains for *Trcul1*, *Trskp1*, and *Trrbx1* with the *tcu1* promoter, respectively, along with a knockout mutant of *Trcsn5*. Whereas repressing *Trskp1* and *Trcul1* but not *Trrbx1* significantly impaired strain growth, hardly any growth defect was observed with their overexpression in the absence of copper for all the indicated strains except that *Trcul1* overexpression reduced the growth on malt extract plates. Deletion of *Trcsn5* had no observable effect on growth (S8A and S8B Fig). Extracellular *p*NPC hydrolytic activity under cellulase-inducing conditions decreased by approximately 75% with *Trcul1* and *Trskp1* repression as well as deletion of *Trcsn5*, but by only 20% when repressing *Trrbx1* (S8C Fig). These findings underscore the important regulatory roles of a putative nuclear *T. reesei* SCF complex in fungal growth and cellulase gene expression.

### Cre1 is identified as a direct interacting substrate for TrFwd1

To investigate whether the altered transcription of *xyr1* or other relevant transcriptional factors contributes to the observed changes in cellulase gene expression with *Trubc4* repression or *Trfwd1* deletion, quantitative RT-PCR analyses were performed and revealed that the transcript levels of either *xyr1* or the other main repressor genes including *cre1* and *ace1* remained largely unchanged under cellulase-inducing conditions (S9A Fig), suggesting that the putative ubiquitination cascade may not act by altering the transcription of these core regulators, but rather target one of them to modulate its function.

**Fig 5:**
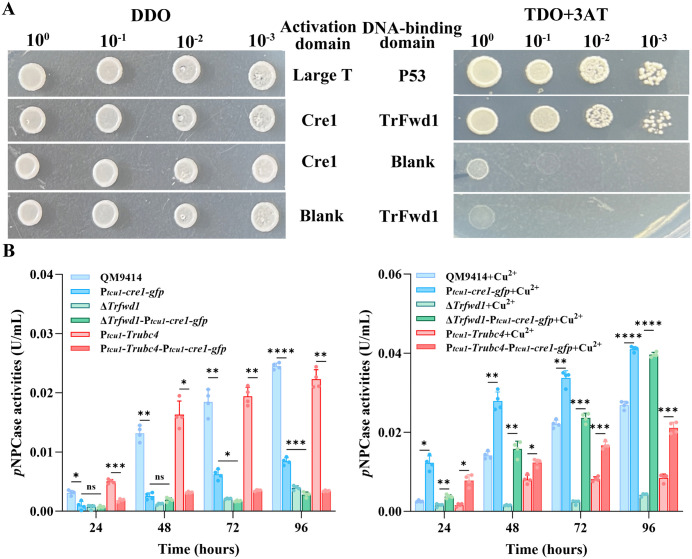
Cre1 is a regulatory target of the TrUbc4-TrFwd1-mediated ubiquitination cascade. **(A)** Protein interaction analysis of TrFwd1 and Cre1 using yeast two-hybrid assay. Yeast cells harboring the indicated combinations of plasmids were plated on TDO lacking leucine, tryptophan, and histidine, and supplemented with 0.5 mM 3-AT. The P53/Large T combinations were used as positive. Tenfold serially diluted cell cultures were inoculated on each spot. **(B)** Extracellular *p*NPC hydrolytic activity of QM9414, the Δ*Trfwd1* or the *Trubc4*-repressed strains with their endogenous *cre1* replaced by a *tcu1* promoter driving *cre1-gfp* (P_*tcu1*_*-cre1-gfp,* Δ*Trfwd1-*P_*tcu1*_*-cre1-gfp,* and P_*tcu1*_*-Trubc4*-P_*tcu1*_*-cre1-gfp*) cultured on 1% Avicel, with or without copper irons to control the expression of *cre1-gfp*. Data are represented as mean ± SD. ^ns^P > 0.05, ^*^P < 0.05, ^**^P < 0.01, ^***^P < 0.001, ^****^P < 0.0001.

Given the well-documented implication of various components involved in putative ubiquitination and de-ubiquitination targeting CreA to control CCR in *Aspergillus* [49,50,52,53], we set out to focus on *T. reesei* Cre1. A yeast two-hybrid assay was first employed to test possible physical interactions between TrFwd1 and several important transcriptional factors including Xyr1, Cre1, or Ace1. Whereas TrFwd1 did not seem to interact with Xyr1 or Ace1, a specific interaction was observed with Cre1 (Figs 5A, S9B and S9C), suggesting that Cre1 is a potential ubiquitination substrate of the identified SCF^TrUbc4-TrFwd1^ complex.

To elucidate the regulatory role of TrFwd1 targeting Cre1 in *T. reesei*, we constructed a C-terminal GFP-tagged Cre1 strain (P_*tcu1*_*-cre1-gfp*) under the control of *tcu1* promoter by replacing the endogenous *cre1* locus. Growth assays revealed that whereas overexpression of *cre1* without copper exerted no discernible impact, repressing *cre1* expression with copper significantly impaired both mycelial expansion and conidiation (S10A Fig), a phenotype similar to the knockout of *cre1* [60]. Unlike the effect on growth, *cre1* overexpression resulted in an approximately 70% reduction in the extracellular cellulase activity relative to QM9414 whereas *cre1* repression led to a significant increase (60%) in cellulase activity (Fig 5B). These results verified that Cre1-GFP was equally functional in mediating cellulase gene repression in *T. reesei*. When *Trfwd1* was simultaneously deleted in P_*tcu1*_*-cre1-gfp*, the obtained Δ*Trfwd1-*P_*tcu1*_*-cre1-gfp* strain exhibited a further reduction in extracellular cellulase activity with Cre1 overexpression upon induction by 1% Avicel, implicating that the absence of TrFwd1 exacerbates the repressive effect of Cre1. In contrast, *cre1* repression fully restored the cellulase activity to the control level, which was even 60% higher at 96 h of induction. Similar phenotypic characteristics regarding the induced cellulase production were observed when both the expression of *cre1* and *Trubc4* were controlled by the *tcu1* promoter. Notably, the simultaneous repression of *cre1* and *Trubc4* with copper resulted in a significant recovery of the enzymatic activity up to 80% of the control level. Together these data suggest that TrUbc4-TrFwd1-mediated ubiquitination likely functions to antagonize Cre1-mediated repression.

To test the effect of the putative de-ubiquitination pathway on Cre1, we investigated the role of *T. reesei* Cre2, a deubiquitinating enzyme homolog of CreB that is thought to antagonize the ubiquintination of CreA [37,50,51]. Deletion of *cre2* in QM9414 resulted in an approximately 20% increase in extracellular *p*NPC hydrolytic activity (S10B Fig). Of note, the absence of *cre2* in Δ*Trfwd1* partially restored extracellular *p*NPC hydrolytic activity, reaching approximately 60% of that in QM9414. Altogether, these results indicate that there exists a strong genetic interaction between TrFwd1 and Cre2 ubiquitylating modification of Cre1 to tightly control the induced cellulase gene expression in *T. reesei.*

### TrUbc4 and TrFwd1 mediate Cre1 ubiquitination that is required for CCR relief

**Fig 6:**
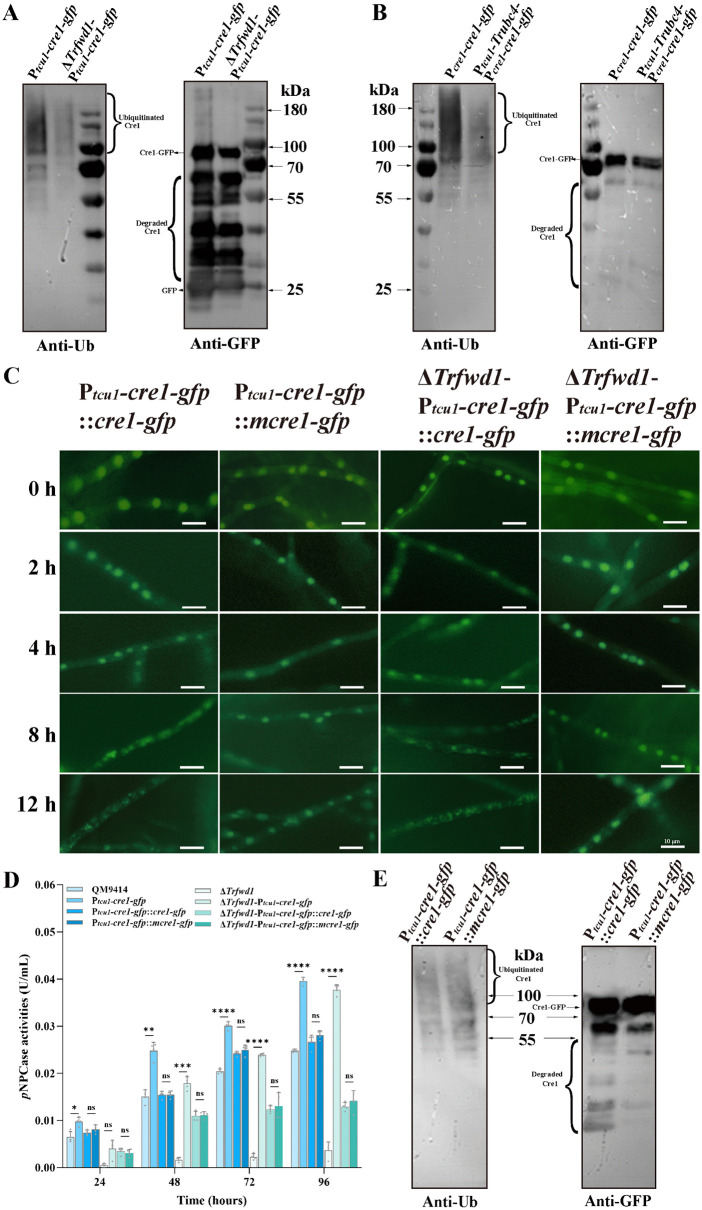
Ubiquitination of Cre1 mediated by TrUbc4/TrFwd1 and the nuclear accumulation of Cre1 had no effect on its function. **(A)** Western blot analysis of Cre1-GFP (predicted molecular weight 71 kDa) was performed on immunoprecipitates obtained from mycelial proteins of P_*tcu1*_*-cre1-gfp* and ΔTr*fwd1-*P_*tcu1*_*-cre1-gfp* strains cultured on 1% Avicel for 2 h. The blots were subsequently probed with an anti-ubiquitin antibody and an anti-GFP antibody, respectively. **(B)** Western blot analysis of Cre1-GFP (predicted molecular weight 71 kDa) was performed on immunoprecipitates obtained from mycelial proteins of P_*cre1*_*-cre1-gfp* and P_*tcu1*_*-Trubc4-*P_*cre1*_*-cre1-gfp* strains cultured on 1% Avicel for 2 h with copper being added to repress *Trubc4*. The blots were subsequently probed with an anti-ubiquitin antibody and an anti-GFP antibody, respectively. **(C)** Fluorescence microscopy observation of the subcellular localization of the P_*tcu1*_-*cre1*-*gfp*::*cre1-gfp*, P_*tcu1*_-*cre1*-*gfp*::*mcre1-gfp,* Δ*Trfwd1-*P_*tcu1*_-*cre1-gfp*::*cre1-gfp* and Δ*Trfwd1-*P_*tcu1*_-*cre1-gfp*::*mcre1-gfp* strains upon transition from 1% glucose to 1% Avicel conditions with copper being added to repress the endogenous *cre1*. **(D)** Extracellular *p*NPC hydrolytic activity of the P_*tcu1*_-*cre1*-*gfp*::*cre1-gfp*, P_*tcu1*_-*cre1*-*gfp*::*mcre1-gfp,* Δ*Trfwd1-*P_*tcu1*_-*cre1-gfp*::*cre1-gfp* and Δ*Trfwd1-*P_*tcu1*_-*cre1-gfp*::*mcre1-gfp* strains cultured on 1% Avicel with copper being added to repress the endogenous *cre1*. **(E)** Western blot analysis of Cre1-GFP and mCre1-GFP was performed on immunoprecipitates obtained from mycelial proteins of P_*tcu1*_-*cre1*-*gfp*::*cre1-gfp* and P_*tcu1*_-*cre1*-*gfp*::*mcre1-gfp* strains cultured on 1% Avicel for 2 h with copper being added to repress the endogenous *cre1*. The blots were subsequently probed with an anti-ubiquitin antibody and an anti-GFP antibody, respectively. Data are represented as mean ± SD. ^ns^P > 0.05, *P < 0.05, **P < 0.01, ***P < 0.001, ****P < 0.0001.

To investigate whether Cre1 was indeed ubiquitinylated by an SCF complex integrating TrFwd1, mycelial lysates from the P_*tcu1*_*-cre1-gfp* and ΔTrFwd1*-*P_*tcu1*_*-cre1-gfp* strains cultured for 2 h of 1% Avicel induction were immunoprecipitated with GFP-Trap beads followed by western blot analysis using anti-GFP and anti-ubiquitin antibodies, respectively. The results revealed that Cre1 was apparently ubiquitylated and this ubiquitination was markedly reduced in the absence of *Trfwd1* (Fig 6A). To check the role of TrUbc4 on Cre1 ubiquitination, we fused GFP tag directly at the C-terminus of the endogenous *cre1* in QM9414 (generating P_*cre1-*_*cre1-gfp*) and P_*tcu1*_*-Trubc4* (generating P_*tcu1*_*-Trubc4-*P_*cre1-*_*cre1-gfp*), respectively (S11A and S11B Fig). Similar reduction in the ubiquitination of Cre1 was observed with *Trubc4* repression (Fig 6B), implicating that TrUbc4 likely functions together with TrFwd1 in mediating Cre1 ubiquitination.

Previous studies have suggested a scenario wherein the ubiquitination of CreA may directly affect its function by promoting its nuclear export to the cytoplasm followed by its degradation [47]. To exclude the possibility that nuclear export is required for the relief of Cre1-mediated CCR, we sought to examine whether forced nuclear retention of Cre1 by blocking its nuclear export influenced cellulase gene expression. A putative nuclear export signal (NES) sequence analogous to the *A. oryzae* CreA were identified in Cre1 (S12A Fig). Simultaneous mutations of two highly conserved residues (L338 and L346) to alanine in NES of *A oryzae* CreA have been reported to result in its substantial nuclear retention and significantly extended half-life even under maltose-inducing conditions [61]. The corresponding mutations were first introduced in Cre1 (mCre1-gfp), which was then integrated at the *pyr4* locus of the P_*tcu1*_-*cre1-gfp* and Δ*Trfwd1-*P_*tcu1*_-*cre1-gfp* strains, respectively, to generate P_*tcu1*_-*cre1*-*gfp*::*mcre1-gfp* and Δ*Trfwd1-*P_*tcu1*_-*cre1-gfp*::*mcre1-gfp* expressing mCre1 from the *cre1* promoter. Corresponding strains expressing WT Cre1 were similarly constructed to obtain P_*tcu1*_-*cre1*-*gfp*::*cre1-gfp* and Δ*Trfwd1-*P_*tcu1*_-*cre1-gfp*::*cre1-gfp*, respectively, as controls. While there was hardly any difference in the growth between the mutant and WT Cre1 control strains on either glucose or malt extract media plates with addition of copper ion to repress the endogenous *cre1* expression (S12B Fig), GFP fluorescence displayed a consistent nuclear localization of both WT Cre1 and mCre1 in *T. reesei* conidia germinated on either glucose or Avicel with copper to repress the endogenous *cre1* (S12C Fig). Further analysis of potential dynamic changes in subcellular localization after mycelia transfer from glucose to Avicel revealed that the nuclear fluorescence signal noticeably diminished for Cre1-GFP after 8 h of Avicel induction and became marginally detectable by 12 h. In contrast, mCre1-GFP maintained strong nuclear fluorescence throughout the induction period regardless of the presence or absence of TrFwd1 (Fig 6C).

Further determination of the extracellular cellulase activity of the P_*tcu1*_-*cre1*-*gfp*::*mcre1-gfp* strain revealed that the persistent nuclear retention of Cre1 had no apparent effect on the induced cellulase biosynthesis compared with the similarly expressed WT Cre1 in P_*tcu1*_-*cre1*-*gfp*::*cre1-gfp* (Fig 6D). However, this induced cellulase gene expression with accumulated mCre1-GFP still required TrFwd1 since its absence displayed a 50% reduction in the extracellular hydrolytic activity. Analysis of Cre1 ubiquitination showed no significant difference in the ubiquitination smear intensity between mCre1-GFP and Cre1-GFP (Fig 6E), implicating that the nuclear-accumulated mutant Cre1 is still capable of being ubiquitinated. Taken together, these results implicate that while ubiquitination of Cre1 may facilitate its nuclear export and subsequent degradation, its export from the nucleus may not be a prerequisite for CCR relief since enhanced nuclear accumulation of Cre1 did not deteriorate Cre1-mediated repression with functional TrFwd1.

### Identification of Cre1 K361 as a major site for targeted ubiquitination by the SCF-TrFwd1 complex

**Fig 7:**
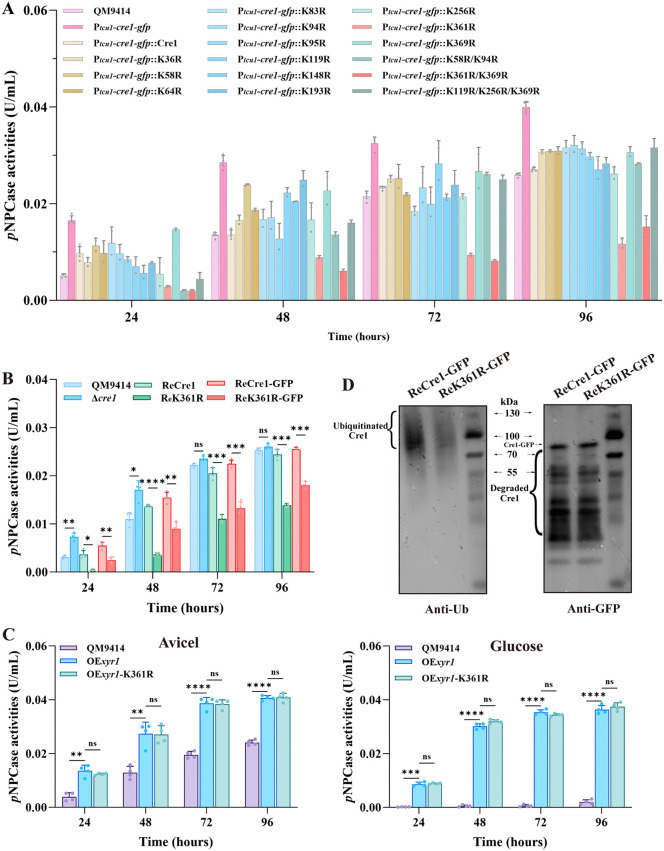
Cre1 K361 represents a key site for ubiquitination to regulate its control over cellulase gene expression. **(A)** Extracellular *p*NPC hydrolytic activity cultured on 1% Avicel with copper being added to repress the endogenous *cre1* while allowing only the expression of Cre1 mutants. Data were from two replicates. **(B)** Extracellular *p*NPC hydrolytic activity of the ReK361R strain with *xyr1* overexpression driven by the *tcu1* promoter (OE*xyr1*-ReK361R) cultured on 1% Avicel (left) or 1% glucose (right). **(C)** Extracellular *p*NPC hydrolytic activity of the Δ*cre1* deletion strain, the corresponding strains complemented with wild-type Cre1 (ReCre1) or the K361R mutant (ReK361R), and a C-terminal GFP-tagged version (ReCre1-GFP and ReK361R-GFP) upon 1% Avicel. **(D)** Western blot analysis of Cre1-GFP was performed on immunoprecipitates obtained from mycelial proteins of ReCre1-GFP and ReK361R-GFP strains cultured on 1% Avicel for 2 h. The blots were subsequently probed with an anti-ubiquitin antibody and an anti-GFP antibody, respectively. Data are represented as mean ± SD. ^ns^P > 0.05, ^*^P < 0.05, ^**^P < 0.01, ^***^P < 0.001, ^****^P < 0.0001.

Cre1 contains 12 lysine (K) residues as potential sites of ubiquitination. To pin down the exact site that is ubiquitylated by the TrUbc4-TrFwd1 enzyme cascade, we individually or combinatorially substituted these lysine for arginine and introduced the respective point mutant as well as WT Cre1 into P_*tcu1*_-*cre1*-*gfp*. All the obtained strains would express the mutant Cre1 from the *pyr4* locus under the control of the *cre1* promoter while its endogenous *cre1* was repressed with copper. Comparing the extracellular *p*NPC hydrolytic activity revealed that while most mutants just behaved like P_*tcu1*_-*cre1*-*gfp*::Cre1 and displayed intermediate cellulase activities between the control and the *cre1* repressed strain, P_*tcu1*_-*cre1*-*gfp*::K361R/K369R and P_*tcu1*_-*cre1*-*gfp*::K361R mutants stood out with significantly compromised cellulase activities that were only 50%-60% of the control level (Fig 7A), indicating an important role of K361 in Cre1 modulating the induced cellulase gene expression. Of note, unlike the growth of the *cre1* repressed control strain, P_*tcu1*_-*cre1*-*gfp*::K361R/K369R and P_*tcu1*_-*cre1*-*gfp*::K361R mutants hardly constrained mycelial growth and exhibited comparable colony expansion to P_*tcu1*_-*cre1*-*gfp*::Cre1 on solid glucose and malt extract media supplemented with copper (S13A Fig), indicating the targeted mutations specifically affect cellulase production while maintaining other functions of Cre1 in supporting hyphal growth and conidiation.

To verify the important role of K361 by avoiding any interference from the leaky expression of the endogenous *cre1* resulting from the *tcu1* promoter, the K361R mutant was introduced into a *cre1* knockout strain. Although Δ*cre1* showed consistent phenotypic defects regarding impaired colony expansion and conidiation with the *cre1* repressed strain (S13B Fig), the complete absence of Cre1 only exhibited a much moderate increase in cellulase production during an early induction period (48 h) (Fig 7B). Complementation of Δ*cre1* with either the WT or the K361R mutant Cre1 almost fully corrected the growth defect (S13B Fig). However, unlike ReCre1, ReK361R failed to initiate an efficient cellulase and xylanase biosynthesis on induction and resulted in a significant reduction in an extracellular *p*NPC and xylan hydrolytic activity (Figs 7B and S2D). These results are consistent with the phenotypic characteristics obtained in the endogenous *cre1* repressed background, confirming the critical role of K361 in Cre1 function. Similar to deletion of *Trfwd1* or repression of *Trubc4*, overexpression of Xyr1 fully restored cellulase biosynthesis in the ReK361R mutant (Fig 7C).

To further investigate whether K361R mutation affects the ubiquitination status of Cre1, GFP-tagged Cre1 (ReCre1-GFP) and K361R (ReK361R-GFP) were expressed in Δ*cre1*, respectively. Both strains behaved almost the same as their untagged counterparts regarding growth and cellulase production (Figs 7C and S13B). The moderate increase in the extracellular cellulase activity in ReK361R-GFP compared with ReK361R suggests a potential interference from GFP with Cre1 function [46]. Notwithstanding this, IP followed by western blot analysis showed significantly reduced ubiquitin modification of K361R-GFP compared to the Cre1-GFP control (Fig 7D), a phenotypic defect that exactly coincides with that caused by the absence of TrUbc4 or TrFwd1. It should be noted that K361R-GFP modification does not seem to impact Cre1 degradation despite the reduced ubiquitination. Altogether these results support the idea that K361 represents a critical site for Cre1 ubiquitination that is dependent on the TrUbc4-TrFwd1 pathway which plays an important role in derepressing Cre1 to facilitate the successful induced cellulase gene expression.

### Ubiquitination of Cre1 diminishes its occupancy on cellulase gene promoters and relieves its antagonizing effect on Xyr1’s binding

**Fig 8:**
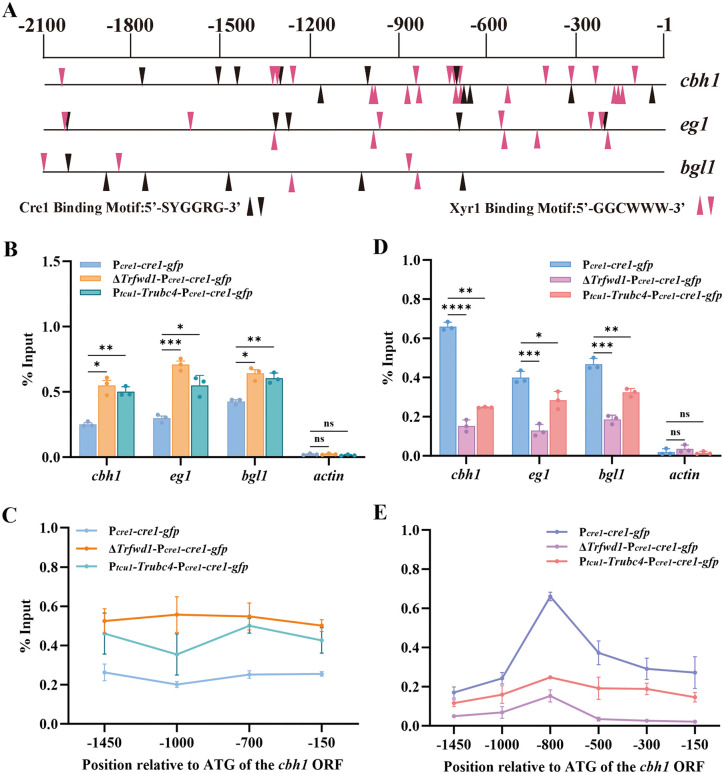
*Trubc4* repression or *Trfwd1* deletion enhanced Cre1 recruitment while reducing Xyr1 occupancy at cellulase gene promoters by ChIP analyses. **(A)** Schematic diagram of the predicted binding sites for Xyr1 and Cre1 on cellulase gene promoters. The numbers are the nucleotide positions relative to the start codon ATG. **(B)** ChIP analyses of Cre1 occupancy on *cbh1*, *eg1*, *bgl1*, and *actin* promoters in *Trfwd1*-deleted strain (Δ*Trfwd1-P*_*cre1*_*-cre1-gfp*) and *Trubc4*-repressed strain (P_*tcu1*_-*Trubc4-P*_*cre1*_*-cre1-gfp*) supplemented with copper. The analyzed promoter regions include P_*cbh1*_ (-604 to -796), P_*eg1*_ (-162 to -323), and P_*bgl1*_ (-1817 to -2123), respectively. **(C)** ChIP analyses to provide an overview of Cre1 occupancy over the *cbh1* promoter in *Trfwd1*-deleted strain (Δ*Trfwd1-P*_*cre1*_*-cre1-gfp*) and *Trubc4*-repressed strain (P_*tcu1*_-*Trubc4-P*_*cre1*_*-cre1-gfp*) supplemented with copper. The analyzed regions include *cbh1*-p1 (-46 to -255), *cbh1*-p2 (-604 to -796), *cbh1*-p3 (–923 to -1088), and *cbh1*-p4 (-1325 to -1533). **(D)** ChIP analyses of Xyr1 occupancy on *cbh1*, *eg1*, *bgl1*, and *actin* promoters in *Trfwd1*-deleted strain (Δ*Trfwd1-P*_*cre1*_*-cre1-gfp*) and *Trubc4*-repressed strain (P_*tcu1*_-*Trubc4*-*P*_*cre1*_*-cre1-gfp*) supplemented with copper. The analyzed promoter regions includes P_*cbh1*_ (-698 to -867), P_*eg1*_ (-162 to -323), and P_*bgl1*_ (-1817 to -2123), respectively. **(E)** ChIP analyses to provide an overview of Xyr1 occupancy over the *cbh1* promoter in *Trfwd1*-deleted strain (Δ*Trfwd1-P*_*cre1*_*-cre1-gfp*) and *Trubc4*-repressed strain (P_*tcu1*_-*Trubc4-P*_*cre1*_*-cre1-gfp*) supplemented with copper. The analyzed regions include *cbh1*-p1 (-46 to -255), *cbh1*-p2 (-235 to -418), *cbh1*-p3 (-399 to -579), *cbh1*-p4 (–698 to -867), *cbh1*-p5 (–923 to -1088), and *cbh1*-p6 (–1325 to -1533). The numbers within brackets are the nucleotide position relative to the start codon ATG. No significant difference (*t* test *P* > 0.05 [n.s.]) was observed for Cre1 or Xyr1 occupancy on cellulase gene or *actin* promoters. All the mycelia mentioned above were collected after induction on 1% Avicel for 2 h. Data are represented as mean ± SD. ^ns^P > 0.05, ^*^P < 0.05, ^**^P < 0.01, ^***^P < 0.001, ^****^P < 0.0001.

Considering that enhancing nuclear accumulation of Cre1 did not deepen CCR, we wondered whether TrFwd1-mediated ubiquitination of Cre1 is able to modulate its binding to target gene promoters. Quite a few Cre1 DNA-binding motifs (5’-SYGGRG-3’) were distributed along the three main cellulase gene promoters (*cbh1*, *eg1*, and *bgl1*), alternating with Xyr1 binding sites (5’-GGCWWW-3’) that are especially highly enriched in the *cbh1* promoter (Fig 8A). To gain an overview of Cre1 recruitment to the various cellulase gene promoters, chromatin immunoprecipitation (ChIP)-qPCR assays were performed to investigate their occupancy by Cre1 with Cre1-GFP expressed in QM9414, P_*tcu1*_-*Trubc4* and Δ*Trfwd1*, respectively, after induction with 1% Avicel (S11A and S11B Fig). As shown in Figs 8B and 8C, a significantly higher enrichment of Cre1 was observed on the respective promoter regions either in the absence of *Trfwd1* or with repression of *Trubc4* compared to the control strain.

To investigate whether the increased Cre1 occupancy interferes with the binding of the major transcriptional activator Xyr1, similar ChIP assays with an Xyr1-specific antibody were performed. In contrast with Cre1, whereas a relatively higher recruitment of Xyr1 occurred on cellulase gene promoters upon 1% Avicel induction, the enrichment signals for Xyr1 were significantly reduced with compromised ubiquitination of Cre1 (Figs 8D and 8E). As expected, no significant enrichment of Cre1 or Xyr1 was detected on the actin promoter. Altogether these data suggest that the identified TrUbc4–TrFwd1 pathway as well as Cre1 K361 are involved in modulating the promoter binding ability of Cre1, which is critical for relieving its interference with the efficient Xyr1 binding to activate cellulase gene expression.

**Fig 9:**
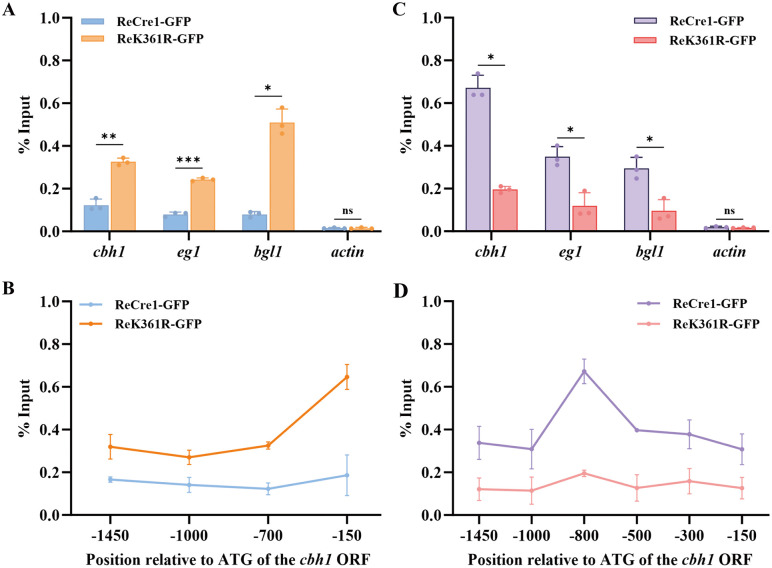
Cre1-K361R increased Cre1 recruitment while reducing Xyr1 occupancy at cellulase gene promoters. **(A)** ChIP analyses of Cre1 occupancy on *cbh1*, *eg1*, *bgl1*, and *actin* promoters in the ReK361R-GFP strain. The analyzed promoter regions includes P_*cbh1*_ (-604 to -796), P_*eg1*_ (-162 to -323), and P_*bgl1*_ (-1817 to -2123), respectively. **(B)** ChIP analyses to provide an overview of Cre1 occupancy over the *cbh1* promoter in the ReK361R-GFP strain. The analyzed regions include *cbh1*-p1 (-46 to -255), *cbh1*-p2 (-604 to -796), *cbh1*-p3 (–923 to -1088), and *cbh1*-p4 (-1325 to -1533). **(C)** ChIP analyses of Xyr1 occupancy on *cbh1*, *eg1*, *bgl1*, and *actin* promoters in the ReK361R-GFP strain. The analyzed promoter regions includes P_*cbh1*_ (-698 to -867), P_*eg1*_ (-162 to -323), and P_*bgl1*_ (-1817 to -2123), respectively. **(D)** ChIP analyses to provide an overview of Xyr1 occupancy over the *cbh1* promoter in the ReK361R-GFP strain. The analyzed regions include *cbh1*-p1 (-46 to -255), *cbh1*-p2 (-235 to -418), *cbh1*-p3 (-399 to -579), *cbh1*-p4 (–698 to -867), *cbh1*-p5 (–923 to -1088), and *cbh1*-p6 (–1325 to -1533). The numbers within brackets are the nucleotide position relative to the start codon ATG. No significant difference (*t* test P > 0.05 [n.s.]) was observed for Cre1 or Xyr1 occupancy on cellulase gene or *actin* promoters. All the mycelia mentioned above were collected after induction on 1% Avicel for 2 h. Data are represented as mean ± SD. ^ns^P > 0.05, ^*^P < 0.05, ^**^P < 0.01, ^***^P < 0.001, ^****^P < 0.0001.

To further corroborate the effect of ubiquitination on Cre1 occupancy on cellulase gene promoters, ReK361R-GFP mutant was checked for its binding to relevant promoters by ChIP assays. The results revealed that ReK361R-GFP was significantly more enriched on the corresponding promoter regions compared to the Cre1-GFP control upon 1% Avicel induction (Figs 9A and 9B). Similar to what was observed in P_*tcu1*_-*Trubc4* and Δ*Trfwd1*, the increase in Cre1 occupancy was accompanied by a concomitant reduction in Xyr1 recruitment at the *cbh1*, *eg1*, and *bgl1* promoters in the ReK361R-GFP strain (Figs 9C and 9D). Although a direct competition between binding of these important transcription factors can not be deduced, these findings implicate that K361R mutation may impair the ubiquitin modification of Cre1 which leads to its more persistent binding to DNA, but consequently impedes Xyr1 access to the cellulase gene promoters.

## Discussion

In the present study, we provided multifaceted evidence supporting the existence of a nuclear ubiquitination cascade consisting of the ubiquitin-conjugating enzyme, TrUbc4, and an F-box protein, TrFwd1, that acts to ubiquitylate *T. reesei* Cre1. Ubiquitination of Cre1 compromises its binding to gene promoters leading to the relief CCR under cellulase-inducing conditions. Unlike *S. cerevisiae* Mig1, these findings thus dissect a previously speculated molecular mechanism controlling fungal CCR and underscore the diversity of post-translational modifications in regulating similar eukaryotic cellular processes.

Although TrUbc4 was unexpectedly identified in a yeast one-hybrid screen with the major cellulase gene *cbh1* promoter as the bait, the importance of TrUbc4 in modulating the induced cellulase gene expression was unequivocally established through mutational analysis. The unexpected identification of TrUbc4 as a *cbh1* promoter binding protein indeed does not conform to the fact that no evidence for any direct DNA binding by E2 enzymes has been reported. One might speculate that the observed weak signal in the yeast one-hybrid screen may very likely result from an accidental interaction between TrUbc4 and the *cbh1* promoter. Considering that the involved promoter region (-474 bp to -838 bp) is rich in binding sites for transcriptional factors including Cre1 (5’-SYGGRG-3’), an alternative explanation is that TrUbc4 was indirectly recruited to the promoter via other unidentified DNA binding proteins in yeast. Notwithstanding these, the importance of TrUbc4 in modulating the induced cellulase gene expression was unequivocally established through mutational analysis. The absolute functional requirement of the catalytic Cys85 further implicates that a potentially active ubiquitination cascade involving TrUbc4 participates in this regulatory aspect. It should be also noted that our systematic knockout attempts to target all other E2 enzyme genes in *T. reesei* revealed that *Trubc4* was the only gene for which a null mutant could not be obtained, providing strong genetic evidence for its essential role in fungal growth.

Given the unusual disproportionate number of E2s versus E3s and therefore the possible pairing of one E2 with multiple E3s, dissecting specific E2-E3 as well as E3-substrate pairs always presents a formidable task. While previous studies have delineated that Phe62 (UbcH5c) and Ala96 (UbcH5b) in human UBC4 family are important for its interaction with RING E3 [57,58], our mutagenesis study verified that the corresponding F62A/A96D double mutant of TrUbc4 significantly compromised the ability of TrUbc4 to support the induced cellulase gene expression. Together with the observation that a direct interaction exists between TrUbc4 and the Rbx1 RING-finger protein, the results unambiguously hint that TrUbc4 couples with a RING-type E3 ligase to form an active E2 ~ Ub-E3 complex.

The observation that a nuclear-localized F-box protein encoding gene *Trfwd1* largely phenocopied *Trubc4* provided another strong support that a nuclear localized RING-type ubiquitination cascade acts specifically to ensure the efficient cellulase gene expression in *T. reesei*. Further analyses indicated TrFwd1 as an integral component of a putative *T. reesei* SCF ubiquitin ligase (SCF^TrFwd1^) including the scaffold protein Cul1 and the adaptor protein Skp1 as well as multiple subunits of the COP9 signalosome, a known regulator of the SCF complex activity [62,63]. Although prior genetic studies in *A. nidulans* and other filamentous fungi have implicated several factors including the CreB–CreC deubiquitination complex and the CreD–HulA ubiquitin ligase complex in regulating CCR potentially by targeting CreA [49,50,52,53], direct evidence for modification of CreA by ubiquitin has been lacking [64]. Our results for the first time unequivocally showed that instead of CreD and a putative HECT-type E3, an F-box protein TrFwd1 directly interacts Cre1 and mediates its ubiquitination. This fact was further supported by the observation that repression of *cre1* fully rescued the defective induced cellulase production resultant from the absence of TrFwd1. The partial recovery of cellulase activity with the simultaneous repression of *cre1* and *Trubc4* implies that TrUbc4 may participate in additional E3 pathways beyond the SCF^TrFwd1^ complex to effect other regulatory aspects pertaining to cellulase gene expression. An interesting note arising from the characterization of Cre1 was that while knockout of *cre1* did not result in significant changes in cellulase activities except with only a moderate increase during the first 48 h under 1% Avicel induction compared to the parent strain QM9414, downregulating Cre1 expression with a conditionally repressible promoter led to a substantial enhancement (approximately 1.5-fold) in cellulase activities. These results suggest that as a transcriptional repressor, the complete absence of Cre1 is not absolutely associated with the high-level cellulase production. Instead, residual Cre1 likely resultant from the leaky expression appears to be conducive to the efficient biosynthesis of these hydrolytic enzymes. This phenomenon might be attributed to the potential dual role of *T. reesei* Cre1 as reported that certain truncated *cre1* can potentially function as a transcriptional activator [60,65].

Tanaka et al. previously discovered that a C-terminal 20-amino acid sequence of *A.oryzae* CreA is required for proteolytic degradation and specific CCR regulation [61]. However, a potential ubiquitination lysine residue (K396) within this region was not proved to be responsible for the induced degradation of CreA. Here we pinned down K361 of Cre1 as the key ubiquitination site targeted by the SCF^TrFwd1^. K361 (K388 in *A. oryzae* CreA) is another highly conserved lysine residue adjacent to K396 (K369 in *T. reesei*). It would be therefore interesting to see whether K388 is the actual ubiquitination site controlling the stability of CreA in *A. oryzae*. Of note, Cre1 K361R specifically affected cellulase production while the targeted mutation hardly compromised other functions of Cre1 in controlling hyphal growth and conidiation. Given that a wide range of genes have been found to be regulated by deletion of *creA*/*cre1* in *A. nidulans* and *T. reesei* [66–68], it is tempting to hypothesize that Cre1 ubiquitination is a specific response to Avicel or xylan induction and the modification is confined to the regulation of the induced cellulase/xylanase gene expression. It should be also noted that although Cre1 K361R largely phenocopied the absence of *Trfwd1* or the downregulation of *Trubc4*, the mutant still displayed a residual but significant ubiquitination signal, indicating that its modification by ubiquitin was not completely abolished. Considering the multiple lysine residues in Cre1, it is reasonable to deduce that other lysine than K361 may still be prone to ubiquitination, but most probably with lower efficiency by either the SCF^TrFwd1^ complex or even other unidentified E3 ligases. Whether these other modifications than that on K361 also participate in CCR regulation requires further investigation.

Although there was no previous definitive evidence for the ubiquitination of CreA as well as the involved ubiquitylating enzymes, quite a few studies have reported the proteolytic degradation of the CreA protein and its counterparts in *A. nidulans*, *A. oryzae*, and *T. reesei* [46,47,61]. An associated cellular process that has been noticed is the change of its subcellular localization. Whereas convinced evidence for linking the modification of CreA by ubiquitin with the above two processes is still lacking, it has been demonstrated that blocking the nuclear export of CreA of *A. oryzae* by mutating its NES also prevents degradation of CreA upon maltose induction [61]. On the other hand, contradictive evidence existed showing that although the nuclear export of CreA was not blocked by disruption of the AMPK gene *snfA*, it was obviously stabilized by *snfA* disruption [48]. These results suggest that besides nuclear export regulation, multiple mechanisms are involved in the control of CreA stability and thus its function. Building on these regulatory aspects, our results otherwise revealed that the ubiquitination of Cre1 facilitates its release from target gene promoters, which is accompanied by the efficient binding of the key transcriptional activator Xyr1 required for the induced cellulase gene expression. This observation is consistent with a previous report showing that the prevention of Mig2 degradation inhibited its release from DNA, resulting in the repression of the galactose-induced gene expression [28]. Of note, even Cre1 was stabilized and accumulated in the nucleus by the NES mutation, it had no apparent effect on cellulase gene expression. Similar results were obtained for *A. oryzae* and *A. nidulans* CreA [47,61]. Altogether, these observations otherwise suggest that nuclear export and degradation of CreA are not prerequisite for the release of CreA bound to the promoter region of carbon catabolite-repressible genes. Whether and how the ubiquitination of Cre1 serve as a signal for nuclear exclusion and subsequent processing for degradation warrants further investigation. It should be also noted that unlike *S. cerevisiae* Gal4 and the transcriptional repressor Ace1 of *P. oxalicum,* which apparently necessitate the UPS to fulfill their respective regulatory functions [27,29], relieving the repressive roles of *T. reesei* Cre1 by ubiquitination represents another example of protein PTMs in modulating transcription although the exact mechanism remains to be dissected.

**Fig 10:**
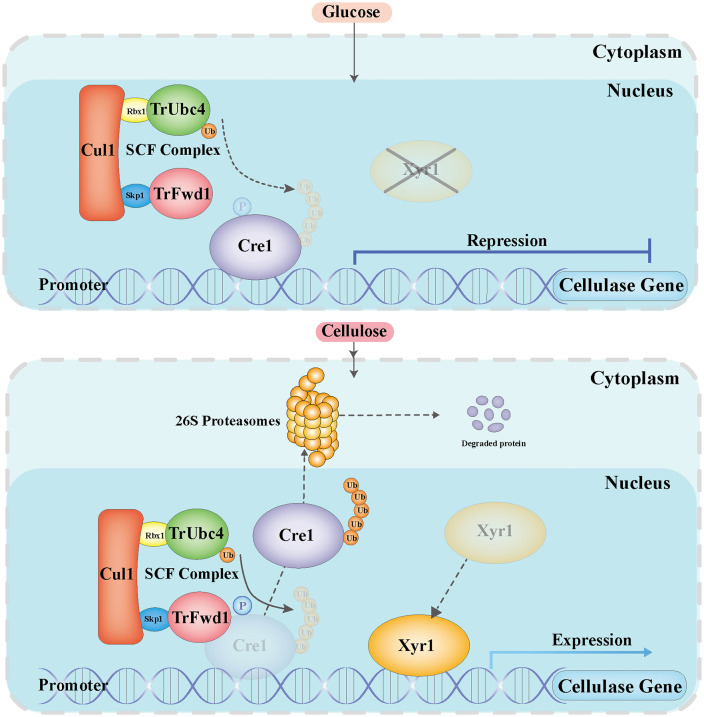
A proposed model illustrating how a nuclear SCF^TrUbc4-TrFwd1^ complex targets Cre1 for ubiquitination to regulate cellulase gene expression.

When targeting a specific protein for ubiquitination, there have been numerous cases wherein phosphorylation of the substrate usually takes place first to provide a recognition signal for E3 ubiquitin ligases to initiate the ubiquitylating cascade. Both *A. nidulans* CreA and *T. reesei* Cre1 have been reported to possess multiple phosphorylation sites [69,70]. Moreover, phosphorylation of Cre1 has been shown to effect its DNA-binding activity in *T. reesei* [71]. José de Assis et al. recently showed that protein kinase GskA simultaneously interacts with *A. nidulans* CreA and the Fbx23 SCF complex, which also interacts with the casein kinase CkiA under derepressing conditions [53]. These observations led to a hypothesis that protein kinase GskA, probably together with CkiA, initially switch on the phosphorylation of the CreA repressor complex, which is then subject to subsequent degradation via ubiquitylation by the Fbx23 E3 ligase SCF complex. Although more mechanistic details regarding the intricate interrelationship among Cre1 phosphorylation, ubiquitination, nucleo-cytoplasmic shuttling, and degradation await for being revealed, our aforementioned results depict a possible working model for fine tuning Cre1 function (Fig 10). Under repressing conditions (e.g., glucose), Cre1 is predominantly nuclear-localized, largely unphosphorylated, and effect CCR to repress cellulase gene expression. Upon induction with cellulose, putative kinases may trigger Cre1 phosphorylation which facilitate s the recruitment of TrFwd1, a component of the SCF E3 ligase complex, to promote Cre1 ubiquitination and its subsequent release from promoters. The ubiquitinated Cre1 may then be exported to the cytoplasm via specific karyopherins and targeted for degradation. Concurrently, Xyr1 is induced, enabling its binding to cellulase gene promoters to activate transcription.

In summary, this study moves beyond the traditional transcriptional regulatory framework by systematically dissecting the involvement of a protein ubiquitination cascade in cellulase gene regulation in *T. reesei*. The identified nuclear ubiquitination pathway specifically targets the transcriptional repressor Cre1 for functional inactivation without an absolute prerequisite for its degradation upon the shift of nutritional cues. This work not only contributes to our understanding of the sophistication and diversity of post-translational control of protein functions in eukaryotic gene transcription, but also provides detailed mechanistic insights into the regulation of CCR in the industrial cellulolytic organism *T. reesei* to help engineer the improved cellulase production*.*

## Materials and methods

### Strains and culture conditions

*T. reesei* QM9414 (ATCC 26921) and Δ*pyr4*, in which the uridine trophic marker gene was deleted in QM9414, were used throughout this work as control and parental strains, respectively. All *T. reesei* strains were maintained on malt extract agar (30 g/L malt extract, 1.5% Agar). For transcription and cellulase production analysis, *T. reesei* strains were pre-grown in 1 L Erlenmeyer flasks on a rotary shaker (200 rpm) at 30°C in 250 mL Mandels-Andreotti (MA) medium with 1% glycerol as the carbon source for 48 h. Mycelia were harvested by filtration and washed twice with medium without carbon source. Equal amounts of mycelia were then transferred to a fresh medium without peptone containing 1% Avicel or other carbon sources as indicated, and incubation was continued for the indicated time period. MA medium was prepared as follows: Na_2_HPO_4_·12H_2_O 17.907 g/L, KH_2_PO_4_ 2 g/L, (NH_4_)_2_SO_4_ 1.4 g/L, Urea 0.3 g/L, Tween-80 0.5 ml/L, Carbon source 1% (w/v). The solution was brought to 1 L with deionized water with pH being adjusted to 5.0 with anhydrous citric acid. The medium was finally sterilized. MgSO_4_·7H_2_O 0.6 g/L, CaCl_2_ 0.6 g/L, and 1 × trace elements were added. Uridine (2.4 g/L) was supplemented when required. Based on the provided formula for the 1000 × trace element stock solution (0.5 g FeSO_4_·7H_2_O, 0.16 g MnSO_4_·H_2_O, 0.14 g ZnSO_4_·7H_2_O, and 0.2 g CoCl_2_·2H_2_O per 100 mL). For pre‑culture with glycerol, peptone (2.0 g/L) was added to MA. Minimal Medium (MM) used for vegetative growth assay was prepared as follows: 20 g glucose, 5 g (NH_4_)_2_SO_4_, and 15 g KH_2_PO_4_ per liter. The pH was adjusted to 5.5 using NaOH. After sterilization, the following sterile stock solutions were added per liter of medium, 4 mL of 250 × MgSO_4_ stock solution (0.61 M), 4 mL of 250 × CaCl_2_ stock solution (1.35 M), and 1 ml of filter-sterilized 1000 × trace element stock solution. All *T. reesei* strains are listed in S4 Table.

*Escherichia coli* DH5α was used for plasmids construction and *E. coli* BL21 (DE3) was used as a host for the production of recombinant proteins. Both strains were cultured on Luria-Bertani medium (1% yeast extract, 2% peptone and 2% NaCl) with a rotary shaker (200 rpm) at 37°C.

*S. cerevisiae* strain Y187 (*MATa*, *ura3-52*, *his3-200*, *ade2-101*, *trp1-901*, *leu2-3*, *112*, *gal4*Δ, *gal80*Δ, *met*^*–*^, *URA3*::*GAL1UAS*–*Gal1*_*TATA*_*–LacZ MEL1*) was used as the host for one-hybrid screening. *S. cerevisiae* strain Y2HGold (*MATa*, *trp1-901*, *leu2-3*, *112*, *ura3-52*, *his3-200*, *gal4*Δ, *gal80*Δ, *LYS2*::*GAL1*_*UAS*_-*Gal1*_*TATA*_-*His3*, *GAL2*_*UAS*_-*Gal2*_*TATA*_-*Ade2 URA3*::*MEL1*_*UAS*_-*Mel1*_*TATA*_ *AUR1*-*C MEL1*) were used for yeast two-hybrid assay. Yeast cells were routinely cultivated at 30°C in YPD medium (1% yeast extract, 2% peptone and 2% glucose). Yeast transformants were cultivated in synthetic complete (SC) medium with appropriate amino acids used for auxotroph selection. All Y2H assay were performed according to the manufacturer’s manual (Clontech-TaKaRa Bio).

### cDNA library and plasmids for yeast-based screen

For preparation of the cDNA library, strain QM9414 mycelia induced on 1% Avicel for 3 h were harvested and mRNAs were extracted using the TRIzol reagent (Invitrogen). A cDNA library was constructed by ligating the reverse transcribed cDNA fragments into the pGADT7 plasmid according to the Matchmaker two-hybrid system manual (Clontech-TaKaRa Bio) [54]. Ten micrograms of *T. reesei* cDNA library DNA was then transformed into the Y187 strain. The bait plasmid used for the yeast one-hybrid screen was constructed by amplifying the AbA (Aureobasidin A) resistance gene *AUR1-C* from *S. cerevisiae* Y2HGold (Clontech-TaKaRa Bio) genomic DNA and inserting it into pRS304 that had been digested with *Xho*I/*Not*I. The *S. cerevisiae gal1* core promoter was then amplified from *S. cerevisiae* genomic DNA and inserted upstream of the *AUR1-C* gene within the *Apa*I and *Xho*I sites to obtain the pRS1 plasmid [54]. The 365 bp *cbh1* promoter region (-474 to -838 bp) was finally amplified from *T. reesei* genomic DNA which was ligated into the pRS1 plasmid digested with *Kpn*I and *Apa*I to obtain the pRS3 bait plasmid. pRS3 was transformed into Y187 after being linearzied with *Lgu*I to obtain the bait yeast strain, which could not grow on plates with 100 ng/mL AbA, indicating that the *cbh1* promoter (-474 to -838 bp) could not drive reporter gene expression by itself.

The yeast two-hybrid assay utilized pGADT7 and pGBKT7 (Clontech-TaKaRa Bio) as cloning vectors, which were digested using *Nde*I and *Eco*RI to clone the respective target genes. The indicated genes encoding proteins for interaction assay were amplified from *T. reesei* cDNA, subsequently digested and ligated into the above plasmids before being transformed into Y2HGold strain.

Yeast transformation was performed using a commercial yeast transformation kit (Clontech-TaKaRa Bio). Briefly, yeast cells were cultured on YPD to a mid-log phase (OD600 = 0.4-0.5), which were then collected and washed twice with sterile water, once with LA buffer (100 mM LiAc, 10 mM Tris–HCl, 1 mM EDTA, pH 7.5), and finally resuspended in 600 μL LA buffer on ice to prepare competent cells. For transformation, 20 μg denatured carrier DNA that was boiled for 5–10 min and chilled on ice, and 5 μg plasmid were mixed with 600 μL competent cells, followed by addition of 2.5 mL LAP buffer (LA buffer supplemented with 40% PEG-3350). After a 45-min incubation at 30 °C with gentle mixing every 15 min, 160 μL DMSO was added. The mixture was heat-shocked at 42 °C for 25 min with intermittent mixing, then cooled on ice for 2 min. To enhance transformation efficiency, cells were finally recovered in 3 mL YPD Plus medium (30 °C, 200 rpm, 90 min), washed once with 4 mL 0.9% NaCl, and resuspended in 4 mL 0.9% NaCl. Transformants were selected on SC plates with appropriate amino acids used for auxotroph selection. The transformant colonies were picked and the harbored plasmids were verified by DNA sequencing after retransformation.

### Plasmids and recombinant *T. reesei* strains construction

All gene knockouts in *T. reesei* were performed by transforming the indicated strains with a gene knockout cassette obtained via the double-joint (fusion) PCR method [72]. Briefly, the upstream homologous arm (~2 kb) of the target gene, a selectable marker gene such as the uridine auxotrophic marker gene *pyr4* or the hygromycin B resistance gene *hph*, and the downstream homologous arm (~2 kb) were PCR fused together to generate the final knockout cassette.

All complemented gene expression in *T. reesei* involved constructing the gene expression cassette into pUC19 [73], followed by linearizing the plasmid with an appropriate restriction enzyme that avoids cutting the expression cassette. Plasmid construction was performed using the one-step T5 exonuclease DNA assembly (TEDA) protocol [74]. All primers used were listed in S5 Table.

To construct a plasmid for conditionally repressing *Trubc4* expression under the control of the copper-responsive promoter P_*tcu1*_, a 2.2 kb coding region starting from the start codon ATG was amplified from genomic DNA of *T. reesei* QM9414, and inserted into the *Asc*I site of the pMDP_*tcu1*_-*pyr4* plasmid [73]. Subsequently, the resulting plasmid was digested with *Hin*dIII and ligated with a 2.0 kb DNA fragment corresponding to the upstream non-coding region of *Trubc4*. The plasmid was then linearized with *Sca*I before being transformed into the the Δ*pyr4* and the OE*xyr1* [75] strains to obtain P_*tcu1*_-*Trubc4* and OE*xyr1*-P_*tcu1*_-*Trubc4*, respectively.

For P_*tcu1*_-controlled expression of GFP-TrUbc4 at the endoenous *Trubc4* locus in *T. reesei*, the coding sequence of *Trubc4* was amplified from the pGADT7-TrUbc4 plasmid, and 1.5 kb downstream of *Trubc4* was amplified from genomic DNA of *T. reesei* QM9414, The resulting PCR products were fused and digested with *Asc*I and *Spe*I, and then ligated into pMDP_*tcu1*_-*gfp*-T_*trpC*_ [76]. Subsequently, the resulting plasmid was digested with *Hin*dIII and ligated with a 2.0 kb DNA fragment corresponding to the upstream non-coding region of *Trubc4*. The resulting plasmids were linearized with *Ssp*I before being transformed into the Δ*pyr4* strain to obtain the P_*tcu1*_-*gfp*-*Trubc4* strain.

To construct strains with targeted expression at the *pyr4* locus, DNA fragments corresponding to approximately 1.8 kb of upstream and 1.4 kb of downstream non-coding regions of the *pyr4* gene were first amplified from genomic DNA of QM9414. These fragments were then fused the *T*_*trpC*_ terminator and the *hph* selectable marker via overlapping PCR. The final PCR product with the up- and downstream homologous arms flanking the the *T*_*trpC*_ terminator and *hph* was ligated into the *Sma*I site of pUC19 to obtain the pUC19-*pyr4::T*_*trpC*_*-hph* plasmid.

To achieve the complemented expression of *Trubc4* at the *pyr4* locus, the *Trubc4* coding sequence and an 1.4 kb *tef1* promoter were amplified from pGADT7-TrUbc4 and QM9414 genomic DNA, respectively, followed by PCR fusion. The fused fragment was subsequently ligated into the *Sgs*I site of the pUC19-*pyr4::T*_*trpC*_*-hph* plasmid to make *Trubc4* under the control of P_*tef1*_. Similarly, the C85A and F62A/A96D TrUbc4 mutant expression cassettes were constructed using the same strategy. The resulting plasmids were linearized with *Sma*I and transformed into the P_*tcu1*_-*Trubc4* strain to obtain the ReUbc4, ReC85A, and ReF62A/A96D strains, respectively.

To express the N-terminal GFP or Myc-His tagged TrFwd1 at the *Trfwd1* locus in *T. reesei*, a two-step assembly strategy was employed. First, a fusion DNA fragment was generated by PCR comprising sequentially the 0.8 kb constitutive P_*gpd1*_ promoter amplified from QM9414 genomic DNA, the GFP or 5 × Myc-6 × His coding sequence, the full-length TrFwd1 or its ΔF-box mutant coding sequence, and an 1.6 kb downstream non-coding region of *Trfwd1*. The obtained DNA fragment was ligated into the *Bam*HI/*Hin*dIII sites of pUC19. Second, a DNA fragment containing an upstream non-coding region of *Trfwd1* and the *pyr4* gene were amplified from QM9414 genomic DNA and fused together with PCR, which was then ligated into the *Eco*RI site of the intermediate plasmid from step one. The final plasmid was linearized with *Eco*105I and transformed into the Δ*Trfwd1* strain to generate the complemented strains including P_*gpd1*_-*gfp*-*Trfwd1*, P_*gpd1*_-*gfp*-*Trfwd1*-ΔF, P_*gpd1*_-*myc*-*his*-*Trfwd1*, and P_*gpd1*_-*myc*-*his*-*Trfwd1*-ΔF, respectively.

To construct plasmids for replacing the *Trcul1*, *Trrbx1*, and *Trskp1* promoters with the P_*tcu1*_ promoter, P_*tcu1*_ and the *pyr4* gene were first amplified from QM9414 genomic DNA. These two DNA fragments were subsequently PCR fused together and ligated into *Bam*HI/*Hin*dIII-digested pUC19 to generate the pUC19-P_*tcu1*_-*pyr4* plasmid. Plasmids for promoter replacement was further constructed using a two‑step cloning strategy. DNA fragments corresponding to the respective coding sequences and 1.5 kb downstream non-coding regions were firstly amplified from QM9414 genomic DNA. The resulting DNA fragments were individually ligated into the *Hin*dIII-digested pUC19-P_*tcu1*_-*pyr4* plasmid. The obtained intermediate plasmids were then digested with *Sgs*I and the corresponding 1.5 kb to 1.9 kb upstream non-coding homologous regions of each target gene were inserted to generate the final targeting plasmids. Each plasmid was linearized with *Ssp*I and transformed into the Δ*pyr4* strain to obtain the P_*tcu1*_‑*Trcul1*, P_*tcu1*_‑*Trrbx1*, and P_*tcu1*_‑*Trskp1* strains, respectively.

To achieve the expression of *cre1-gfp* under the control of the *P*_*tcu1*_ promoter, the respective plasmids were constructed using a two-step cloning strategy. The *cre1* gene along with approximately 1.7 kb of its downstream non-coding region was first amplified from QM9414 genomic DNA and PCR fused with *gfp*. The obtained DNA fragment was then inserted into the *Hin*dIII site of pUC19-P_*tcu1*_-*pyr4*. Subsequently, an 1.6 kb upstream non-coding region of *cre1* was inserted into the *Sgs*I site of the above intermediate plasmid to obtain pUC19- P_*tcu1*_-*cre1-gfp* plasmid, which was then linearized with *Eco*105I before being transformed into the Δ*pyr4* and the Δ*Trfwd1* strains to obtain the P_*tcu1*_-*cre1-gfp,* Δ*Trfwd1-*P_*tcu1*_*-cre1-gfp*, respectively*.* By substituting the *hph* marker for the *pyr4* selection marker in pUC19-P_*tcu1*_-*pyr4*, the similarly modified targeting plasmid was linearized with *Eco*105I and transformed into the P_*tcu1*_*-Trubc4* strain to obtain the P_*tcu1*_*-Trubc4-*P_*tcu1*_*-cre1-gfp* strain.

For targeted integration of the *cre1-gfp* expression cassette to substitute for the endogenous *cre1* locus, a fusion DNA fragment was first assembled by PCR comprising the following six sequential components, an 1.6 kb of its upstream non-coding region including the *cre1* promoter, the *cre1* coding sequence, the *gfp* gene, the T_*trpC*_ terminator, the *hph* marker gene, and an 1.7 kb of the *cre1* downstream non-coding region. The assembled DNA fragment was then ligated into the *Eco*RI-digested pUC19 plasmid to obtain the P_*cre1*_-*cre1*-*gfp* plasmid and the resulting plasmid was linearized with *Lgu*I before being transformed into Δ*pyr4*, Δ*Trfwd1* and P_*tcu1*_-*Trubc4* to obtain the P_*cre1*_-*cre1*-*gfp,* Δ*Trfwd1*-P_*cre1*_-*cre1*-*gfp*, and P_*tcu1*_-*Trubc4*-P_*cre1*_-*cre1*-*gfp* strains, respectively*.*

To generate strains expressing *cre1* or *cre1-gfp* under the control of its own promoter at the *pyr4* locus, the expression cassette comprising the 1.6 kb *cre1* promoter and the *cre1* coding sequence as well as the *gfp* gene (if applicable) was first amplified directly from the previously constructed pUC19-P_*cre1*_-*cre1-gfp* plasmid and fused together. The resultant DNA fragment was subsequently ligated into the *Sgs*I-linearized pUC19-*pyr4::T*_*trpC*_*-hph* plasmid. Following linearization with *Sma*I, the construct was transformed into the P_*tcu1*_*-cre1-gfp or* Δ*cre1* strain. Strains expressing point mutant Cre1 were generated using a similarly strategy.

Transformation of *T. reesei* was performed using PEG-mediated uptake of DNA by protoplast [77]. Briefly, fresh conidia spores of *T. reesei* were inoculated into 100 mL of MM medium and cultured at 30°C with shaking (200 rpm) for 20–24 h to obtain young hyphae suitable for enzymatic digestion. Mycelia were harvested via filtration through a G2 sintered glass funnel and washed three times with SK solution (1.0 M sorbitol, 9.1 mM KH_2_PO_4_, 1.3 mM K_2_HPO_4_) to remove residual medium. The washed mycelia were resuspended in 8 mL of SK solution and incubated with 0.04 g each of snailase (Klontech, Amresco) and lywallzyme (GDMCC, Guangdong) at 30°C for 2-3 h with gentle agitation. Protoplast formation was monitored microscopically before being filtered through a G1 sintered glass funnel and protoplasts were collected by centrifugation at 4,000 × g for 10 min at 4°C, washed twice with 4 mL of STC solution (1.0 M sorbitol, 75 mM CaCl_2_, 34 mM NaCl, 10 mM Tris-HCl, pH 7.5), and finally resuspended in 100 µL of STC. For transformation, plasmid DNA (≥10 µg) was added to the protoplast suspension, followed by the addition of 25 µL of PTC solution (60% PEG4000, 75 mM CaCl_2_, 10 mM Tris-HCl, pH 7.5). The mixture was incubated on ice for 20 min followed by addition of 500 µL of PTC. The reaction mixture was gently mixed and allowed to stand at room temperature for 5 min. Subsequently, 1 mL of STC solution was added, and 200–300 µL aliquots were spread onto regeneration agar plates (MM medium supplemented with 1 M sorbitol). Transformants were selected on minimal medium either for uridine prototroph or for resistance to hygromycin (120 µg/mL). Anchored PCR were used to verify the correct integration events.

### Observation of mycelium by fluorescence microscopy

*T. reesei* conidia spores were inoculated in the MM medium containing 1% glucose for 24 h at 30°C. Mycelia were used directly for microscopic observation or transferred to MA medium containing 1% Avicel induction for different time. After incubation, germlings were fixed on the coverslips using methanol and then stained with 100 μg/ml of DAPI (40,6-diamidino-2-phenylindole dihydrochloride; Beyotime C1002) solution for 5 min. The fluorescence of the mycelium of the recombinant strains was detected with a Nikon Eclipse 80i fluorescence microscope (Nikon, Melville, NY, USA), and images were captured and processed with the NIS-ELEMENTS AR software program.

### The vegetative growth assay

To assay vegetative growth, equal amount of growing mycelia were inoculated on MM agar plates containing glucose or on malt extract agar plates, and incubated at 30°C for 3 days.

For determination of the growth in liquid culture, equal amount of mycelia were transferred to fresh MA with 1% (w/v) glucose as the sole carbon source. At the indicated time points, mycelia cultured on glucose were filtrated on filter paper, dried at 80°C for 48 h, and then weighed.

### Enzyme activity measurements

Cellobiohydrolase and β-glucosidase activities were determined by measuring the amount of p-nitrophenol, using p-nitrophenyl-D-cellobioside (*p*NPC; Sigma) and p-nitrophenyl-β-D-glucopyranoside (*p*NPG; Sigma), respectively, as the substrates. The assays of cellulase activity were carried out in 200 μL of reaction mixture containing 40 μL of culture supernatant and 40 μL of the respective substrate plus 80 μl of 50 mM sodium acetate buffer (pH 4.8) with incubation at 50°C for 30 min. The reaction was stopped by addition of 40 μL of 10% Na_2_CO_3_ (w/v). The amount of p-nitrophenyl is determined by measuring the absorbance at 420 nm. One unit (U) of *p*NPC or *p*NPG activity is defined as the amount of enzyme releasing 1 μmoL of p-nitrophenyl per minute.

CMC (carboxymethyl cellulose sodium salt, Sigma) hydrolytic activity was determined by measuring the released glucose using as the substrate. Briefly, the assay was performed in 120 μL of reaction mixture including 60 μL of 50 mM sodium acetate buffer (pH 4.8) and 60 μL of diluted culture supernatant, and the mixture was then incubated at 50°C for 30 min. The reducing sugar released in the mixture was determined by the 3, 5-dinitrosalicylic acid method with glucose as the standard. One unit (U) of CMC hydrolytic activity is defined as the amount of enzyme releasing 1 μmoL of reducing sugar per minute.

Xylanase activities were determined by measuring the amount of released xylose using xylan as substrate. Briefly, a reaction mixture containing 60 μL of diluted culture supernatant and 60 μL of beechwood xylan (5 g/L) dissolved in 50 mM sodium acetate buffer (pH 4.8) was incubated at 50˚C for 15 min. The reducing sugar released in the mixture was determined using DNS method with xylose as the standard. One unit of enzyme activity was defined as the amount of enzyme capable of releasing 1 μmol of xylose per minute

### Quantitative RT-PCR (qRT-PCR)

Total RNAs were extracted using Trizol reagent (Sangon Biotech, Shanghai, China) and purified using the TURBO DNA-free kit (Ambion, Austin, TX, USA) to eliminate genomic DNA contamination according to the manufacturer’s instructions. Reverse transcription was performed using HiScript Q RT SuperMix for quantitative PCR (qPCR) (Vazyme, Nanjing, China) according to the user protocol. Quantitative PCR was performed using ChamQ Universal SYBR qPCR Master Mix (Vazyme, Nanjing, China) on a Roche LightCycler 96 thermocycler (Roche). Gene expression was analyzed using the comparative ΔΔCT method [78], whereby the target gene’s threshold cycle (CT) value was first normalized to the endogenous control actin within each sample (ΔCT = CT_target_ – CT_actin_), subsequently compared to a calibrator sample to calculate ΔΔCT (ΔΔCT = ΔCT_sample_ – ΔCT_control_), and finally converted to a relative fold-change using the formula 2^^(–ΔΔCT)^.

### DSP Cross-linking and Immunoprecipitation (IP)

The cross-linking procedure for detecting the potential SCF complex in *T. reesei* was performed as follows. Conidia spores were inoculated in the MM medium containing 1% glucose at 30°C for 36 h and the mycelia were transferred to MA medium containing 1% Avicel for a 24-h induction, which were then harvested and washed twice with PBS buffer (137 mM NaCl, 2.7 mM KCl, 10 mM Na_2_HPO_4_, 1.8 mM, pH 7.4). The collected cells were resuspended in PBS buffer containing 1 mM DSP (dithiobis/succinimidylpropionate) and incubated at 30 °C with shaking at 200 rpm for 1 h to allow protein cross-linking. The reaction was subsequently quenched by adding glycine to a final concentration of 50 mM, followed by incubation at room temperature for 15 min with periodic mixing. Finally, the mycelia were washed with PBS buffer, collected by vacuum filtration, snap-frozen in liquid nitrogen, and stored at –80 °C or processed immediately for subsequent experiments.

To perform IP for detection of SCF after crosslinking treatment, the above frozen mycelia were completely ground to a fine powder and then resuspended in lysis buffer (50 mM Tris-HCl, pH 7.4, 120 mM NaCl, 1 mM EDTA, 5% glycerol, 1% Triton X-100, 1% sodium deoxycholate, 0.1% SDS, 40 μM MG132, 20 mM NEM, and a protease inhibitor cocktail). The intracellular protein concentration was determined using the BCA protein assay method with bovine serum albumin (BSA) as the standard. Equal amounts of protein were then used for immunoprecipitation (IP) with ChromoTek GFP-Trap or Myc-Trap Agarose (Proteintech, Wuhan, China) according to the manufacturer’s instructions. Briefly, 60 µL of GFP-Trap or Myc-Trap beads were aliquoted and equilibrated twice with 1200 µL of dilution buffer (10 mM Tris-HCl, pH 7.5, 150 mM NaCl, 0.5 mM EDTA) with centrifugation at 5000 g for 2 min each time. Subsequently, an equal amount of mycelial lysate (concentration-normalized and diluted with dilution buffer as needed) was mixed with the equilibrated beads and incubated with rotation at 4 °C for 3 h. After centrifugation at 5000 g for 2 min, the beads were then washed sequentially with 1.2 mL of dilution buffer containing 20% glycerol, 1 M NaCl, 4 M urea, and 1% Triton X‑100 at 4 °C for 5 min on a rotator. The beads were finally washed with 1.2 mL dilution buffer, and bound proteins were eluted twice with an equal volume of 0.2 M glycine (pH 2.5). The eluate was immediately neutralized with 1 M Tris buffer.

To analyze the ubiquitination of Cre1, conidia spores were inoculated in the MM medium containing 1% glucose at 30°C for 36 h and the mycelia were transferred to MA medium containing 1% Avicel for a 2-h induction. Preparation of total cellular extract, immunoprecipitation with GFP-Trap beads and elution were essentially the same as described above.

### Western blot

Total cellular extract or protein elute from IP was resolved by SDS-PAGE followed by western blotting with specific antibodies for detection. The following antibodies were used, a GFP monoclonal antibody (B-2, catalog #SC-9996, Santa Cruz Biotechnology), a monoclonal anti-ubiquitin antibody (EPR8830, catalog #ab134953, Abcam), and a Myc monoclonal antibody (60003–2-Ig, Proteintech, Wuhan, China).

### Chromatin immunoprecipitation (ChIP) analysis

ChIP assay was essentially as described previously [79]. Briefly, the mycelia were fixed in minimal medium containing 1% formaldehyde for 10 min at 30°C, 200 rpm and then quenched cross-linking by adding 25 mL 1.25 M glycine (pH 7.5) for 5 min [80]. The cross-linked mycelia were ground and resuspended in lysis buffer (50 mM HEPES pH 7.5, 150 mM NaCl, 1 mM EDTA, 0.5% Triton X-100, 0.1% sodium deoxycholate, 0.1% SDS, 1 mM PMSF, protease inhibitors). Crude lysate were further sonicated to obtain an average DNA fragment size of ~500 bp fragments. For each immunoprecipitation assay, 1 ml protein (2 mg/mL) was used and each sample was pre-cleared with protein A/G at 4°C for 5 h, protein A/G pre-coated with 1 mg/mL BSA and 1 mg/mL denatured fish sperm DNA. And then the chromatin immunoprecipitation was performed using 2 μL anti-Xyr1 antibody at 4°C for 5 h followed by with the same amount of Protein A/G for per IP at 4°C for 5 h. The polyclonal anti-Xyr1 antibody was custom-made by immunizing the rabbit with a KHL-conjugated peptide (aa 106–119) of the DNA binding domain of Xyr1. Alternatively, chromatin immunoprecipitation was carried out using ChromoTek GFP-Trap Agarose (Proteintech, Wuhan, China). Following immunoprecipitation and extensive sequential washing steps with low-salt wash buffer (0.1% SDS, 1% Triton X-100, 2 mM EDTA, 150 mM NaCl, 20 mM Tris–HCl, pH 8.0), high-salt wash buffer (0.1% SDS, 1% Triton X-100, 20 mM Tris–HCl, 500 mM NaCl, pH 8.0), and LNDET buffer (0.25 M LiCl, 1% NP40, 1 mM EDTA, 10 mM Tris–HCl, pH 8.0), the immunoprecipitated DNA was eluted with elution buffer (100 mM Tris-HCl, pH 7.8, 10 mM EDTA, 1% SDS, 10 mM NaHCO_3,_ 100 mM NaCl) at 65 °C for 5 hours. The eluted material was then treated with proteinase K at 45 °C for 1 hour. DNA was recovered by phenol-chloroform extraction and ethanol precipitation. The final DNA concentration was determined by absolute quantification using quantitative PCR (qPCR). ChIP–qPCR data were normalized to the corresponding input DNA (incubated with protein G/A beads), and data are presented as percentage of input DNA. ChIP efficiency was calculated as 2^−ΔCt^ × 100%, ΔCt = Ct_Sample_−(Ct_Input,adjusted_). An excel file containing the original numerical data for ChIP-qPCR were included as S6 Table.

### Recombinant protein production in *E. coli*

For the expression of TrUbc4 in *E. coli*, the DNA fragments coding for TrUbc4 were amplified from QM9414 cDNA and ligated into the pGEX4T-1 plasmid after digestion with *Nde*I and *Xho*I to obtain pGEX4T-1-TrUbc4. Then DNA sequencing was performed to confirm. The indicated expression constructs were transformed into CaCl_2_-treated competent *E. coli* BL21 (DE3) cells [81]. *E. coli* strains with the indicated expression constructs were grown at 37°C until they reached an OD600 of 0.5–0.6. IPTG (isopropyl-b-D-thiogalactopyranoside) was added at a final concentration of 1 mM followed by continued incubation for 16 h at 20°C. The induced proteins were purified with Glutathione Sepharose 4B (GE Healthcare), essentially according to the manu facturer’s instructions. The fusion proteins were eluted using 50 mM Tris-HCl (pH 8.0), 10 mM glutathione. All of the protein preparations were stored at −80°C in the presence of 20% (v/v) glycerol.

### *In vitro* ubiquitination assay

Reactions containing 125 nM human E1 activating enzyme UBA1 (abcam, ab207988) and 1 μM E2 conjugating enzyme TrUbc4 were incubated with 1 μg ubiquitin (abcam, ab80760) in ubiquitination buffer (20 mM Tris-HCl pH 7.5, 5 mM MgCl_2_, 0.5 mM DTT, 2 mM ATP) in a reaction volume of 20 μL for different time at room temperature. Samples were stopped with 5 × SDS-PAGE loading buffer to stop the reaction and proceed for western blot with anti-Ub antibody.

### Sequence analysis

Amino acid sequences were obtained from the NCBI database and sequence alignment was performed using ClustalW. Phylogenetic analysis was carried out with MEGA using the neighbor-joining method with 2000 bootstraps [82].

### Statistical analysis

Graphs were produced using Prism (GraphPad Software) showing all individual data points, with each point representing one biological replicate. Figures were assembled in Adobe Illustrator. Statistical significance was determined by two-way analysis of variance (ANOVA) followed by Tukey’s multiple comparisons test from three biological replicates. Data are presented as the mean ± SD of these replicates.

## Supporting information

S1 Table Extracellular cellulase activity of *Trichoderma reesei* E2 homolog mutant strains. (XLSX)

S2 Table Extracellular cellulase activity of *Trichoderma reesei* E3 gene mutant strains. (XLSX)

S3 Table SCF subunits and COP9 components identified by TAP-MS. (XLSX)

S4 Table *Trichoderma reesei* strains used in this study. (XLSX)

S5 Table PCR primers used in this study. (XLSX)

S6 Table The original numerical data for ChIP-qPCR. (XLSX)

S1 Fig Isolation of TrUbc4 as a ubiquitin conjugating enzyme. (A) A cDNA library plasmid (pAS23) containing *Trubc4* cDNA combined with the bait plasmid pRS3 allowed yeast transformants to grow in the presence of Aureobasidin A (AbA). Growth was not observed for the negative controls containing either pGADT7 plus pRS3/pRS1 or pAS23 plus pRS1 without the *cbh1* promoter. Tenfold serially diluted cell cultures were inoculated on each spot. (B) *In vitro* ubiquitination assay of TrUbc4. Recombinant TrUbc4-His was incubated at 30°C for 30 min with or without ATP, Ub, and human E1. Reaction products were immunoblotted using anti-Ub antibody. Bands corresponding to free Ub, polyubiquitinated TrUbc4 (TrUbc4-Ub), and TrUbc4-2Ub, respectively, were indicated.
(TIF)

S2 Fig Repression of *Trubc4* compromised the induced cellulase and xylanase gene expression. (A) Growth of QM9414 and the P_*tcu1*_*-Trubc4* strain on glucose and malt extract plates with or without addition of copper ions. (B) Biomass yield of P_*tcu1*_*-Trubc4* as well as the P_*tcu1*_*-Trubc4* strain complemented with the TrUbc4 (ReTrUbc4) and C85A (ReC85A) mutant at the *pyr4* locus under the control of the *tef1* promoter, in liquid glucose medium with or without copper. (C) Extracellular CMC and *p*NPG hydrolytic activities of P_*tcu1*_*-Trubc4* as well as P_*tcu1*_*-Trubc4* complemented with the TrUbc4 (ReTrUbc4) or C85A mutant (ReC85A) at the *pyr4* locus under the control of the *tef1* promoter, cultured on 1% Avicel with or without copper. (D) Extracellular xylanase activity of P_*tcu1*_*-Trubc4* (with copper), Δ*Trfwd1*, Δ*cre1*, ReCre1, and ReK361R cultured on 0.5% xylan. (E) Quantitative RT-PCR analyses of the transcription of the eg*1* gene and the *bgl1* gene in the P_*tcu1*_*-Trubc4* strain cultured on 1% Avicel induction with adding copper to repress *Trubc4*. Data are represented as mean ± SD. ^ns^P > 0.05, ^*^P < 0.05, ^**^P < 0.01, ^***^P < 0.001, ^****^P < 0.0001.
(TIF)

S3 Fig Phylogenetic analysis of the *T. reesei* E2s and functional validation of GFP-tagged TrUbc4. (A) Phylogenetic analysis of all annotated E2s from *T. reesei* and *S. cerevisiae*. (B) Extracellular *p*NPC hydrolytic activity of the P_*tcu1*_*-gfp-Trubc4* strain cultured on 1% Avicel. Data are represented as mean ± SD. ^ns^P > 0.05, ^*^P < 0.05, ^**^P < 0.01, ^***^P < 0.001, ^****^P < 0.0001.
(TIF)

S4 Fig Identification and domain structure of TrFwd1. (A) Schematic diagram of TrFwd1 domain architecture. (B) Phylogenetic analysis of TrFwd1 and its fungal orthologs.
(TIF)

S5 Fig Deletion of *Trfwd1* affected growth and conidiation. (A) Growth of Δ*Trfwd1* as well as the deletion strain complemented with GFP-TrFwd1 (P_*gpd1*_*-gfp-Trfwd1*) or GFP-TrFwd1 without the F-box domain (P_*gpd1*_*-gfp-Trfwd1-*ΔF) on glucose or malt extract plates. (B) Biomass accumulation of Δ*Trfwd1* as well as the deletion strain complemented with GFP-TrFwd1 (P_*gpd1*_*-gfp-Trfwd1*) or GFP-TrFwd1 without F-box domain (P_*gpd1*_*-gfp-Trfwd1-*ΔF) in liquid glucose medium. (C) Quantitative analysis of the conidiation of Δ*Trfwd1* as well as the deletion strain complemented with GFP-TrFwd1 (P_*gpd1*_*-gfp-Trfwd1*) or GFP-TrFwd1 without F-box domain (P_*gpd1*_*-gfp-Trfwd1-*ΔF) on malt extract solid medium. Data are represented as mean ± SD. ^ns^P > 0.05, ^*^P < 0.05, ^**^P < 0.01, ^***^P < 0.001, ^****^P < 0.0001.
(TIF)

S6 Fig Deletion of *Trfwd1* resulted in a decrease in the induced cellulase gene expression and F-box domain is required for its function. (A) Extracellular *p*NPG, and CMC hydrolytic activities of Δ*Trfwd1* as well as the deletion strain complemented with GFP-TrFwd1 (P_*gpd1*_*-gfp-Trfwd1*) or GFP-TrFwd1 without F-box domain (P_*gpd1*_*-gfp-Trfwd1-*ΔF) cultured on 1% Avicel. (B) Quantitative RT-PCR analyses of the eg*1* gene and the *bgl1* gene in the *Trfwd1* knockout strain under 1% Avicel. (C) Western blot analysis of the expression of GFP-TrFwd1 and GFP-TrFwd1-ΔF with predicted molecular weight of 130 kDa and 125 kDa, respectively, using anti-GFP antibody. Total: total cellular extract; Eluate: protein eluate from GFP-Trap beads. Data are represented as mean ± SD. ^ns^P > 0.05, ^*^P < 0.05, ^**^P < 0.01, ^***^P < 0.001, ^****^P < 0.0001.
(TIF)

S7 Fig Functional verification of the F-box domain with strains expressing TAP-tagged TrFwd1 or TrFwd1-ΔF fused with 5 × Myc-6 × His. (A) Extracellular *p*NPC hydrolytic activity of Δ*Trfwd1* as well as the deletion strain complemented with Myc-His-TrFwd1 (P_*gpd1*_*-myc-his-Trfwd1*) or Myc-His-TrFwd1 without F-box domain (P_*gpd1*_*-myc-his-Trfwd1-*ΔF) cultured on 1% Avicel. (B) Western blot analysis of the expression of Myc-His-TrFwd1 and Myc-His-TrFwd1-ΔF with predicted molecular weight of 112 kDa and 106 kDa, respectively. Protein eluates from the immunoprecipitated Myc-Trap beads were resolved by SDS-PAGE and blotted using anti-Myc antibody. (C) Silver staining of the SDS-PAGE-resolved Myc-His-TrFwd1 protein eluates in (B). The addition of β-ME enables the decrosslinking of proteins. Data are represented as mean ± SD. ^ns^P > 0.05, ^*^P < 0.05, ^**^P < 0.01, ^***^P < 0.001, ^****^P < 0.0001.
(TIF)

S8 Fig Deletion or repression of key SCF subunit genes or the COP9 catalytic subunit compromised growth and the induced cellulase biosynthesis. (A) Growth of the indicated SCF subunit gene mutant strains either with the *tcu1* promoter replacement or gene knockout on glucose and malt extract plates with or without copper. (B) Biomass accumulation of the indicated SCF subunit mutant strains either with the *tcu1* promoter replacement or gene knockout in liquid glucose medium with or without copper. (C) Extracellular pNPC hydrolytic activity of the indicated SCF subunit mutant strains cultured on 1% Avicel. Data are represented as mean ± SD. ^ns^P > 0.05, ^*^P < 0.05, ^**^P < 0.01, ^***^P < 0.001, ^****^P < 0.0001.
(TIF)

S9 Fig Expression of key transcription factors was not affected by *Trfwd1* deletion and no interaction existed between TrFwd1 and Xyr1 or Ace1. (A) Quantitative RT-PCR analyses of the *cre1,xyr1* and *ace1* genes in the *Trfwd1* knockout strain and the P_*tcu1*_*-Trubc4* strain with copper upon 1% Avicel. (B) Protein interaction analysis of TrFwd1 and Xyr1 using yeast two-hybrid assay. (C) Protein interaction analysis of TrFwd1 and Ace1 using yeast two-hybrid assay.Yeast cells harboring the indicated combinations of plasmids were plated on TDO lacking leucine, tryptophan, and histidine. The P53/Large T combinations were used as positive. Tenfold serially diluted cell cultures were inoculated on each spot. Data are represented as mean ± SD. ^ns^P > 0.05, ^*^P < 0.05, ^**^P < 0.01, ^***^P < 0.001, ^****^P < 0.0001.
(TIF)

S10 Fig Repression of *cre1* compromised *T.* *reesei* growth and *cre2* deletion partially recovered the induced cellulase biosynthesis in Δ*Trfwd1.* (A) Growth of QM9414, the Δ*Trfwd1* or the *Trubc4*-repressed strains with their endogenous *cre1* replaced by a *tcu1* promoter-driven *cre1-gfp* (P_*tcu1*_*-cre1-gfp*, Δ*Trfwd1-*P_*tcu1*_*-cre1-gfp*, and P_*tcu1-*_*Trubc4-* P_*tcu1-*_ *cre1-gfp*, respectively), cultured on glucose and malt extract plates with or without copper to control the expression of *cre1-gfp*. (B) Extracellular *p*NPC hydrolytic activity of the *cre2* deletion strains cultured on 1% Avicel. Data are represented as mean ± SD. ^ns^P > 0.05, ^*^P < 0.05, ^**^P < 0.01, ^***^P < 0.001, ^****^P < 0.0001.
(TIF)

S11 Fig C-terminal fusion of *gfp* to the endogenous *cre1* did not affect the induced extracellular *p*NPC hydrolytic activity. (A) Extracellular *p*NPC hydrolytic activity of Δ*Trfwd1-cre1-gfp* cultured on 1% Avicel. (B) Extracellular *p*NPC hydrolytic activity of P_*tcu1*_-*Trubc4*-P_*cre1*_*-cre1-gfp* cultured on 1% Avicel with copper to repress *Trubc4*. Data are represented as mean ± SD. ^ns^P > 0.05, ^*^P < 0.05, ^**^P < 0.01, ^***^P < 0.001, ^****^P < 0.0001.
(TIF)

S12 Fig Mutation of the nuclear export signal (NES) of Cre1 had no effect on growth and its nuclear localization. (A) The nuclear export signal (NES) sequence with residues L338 and L346 of *A. oryzae* CreA corresponding to residues L304 and L310 of *T. reesei* Cre1. (B) Growth of the indicated strains on glucose and malt extract plates with copper being added to repress the endogenous *cre1* while allowing only the expression of the Cre1 mutant. (C) Fluorescence co-localization of the indicated strains on glucose and Avicel, with copper added to repress endogenous *cre1* expression and allow only the expression of Cre1 mutants.
(TIF)

S13 Fig The K361R mutation in Cre1 did not affect mycelia growth. (A) Growth of the indicated strains on glucose and malt extract plates with copper being added to repress the endogenous cre1 while allowing only the expression of Cre1 mutants. (B) Growth of the Δ*cre1* strain, the corresponding strains complemented with wild-type Cre1 (ReCre1) or the K361R mutant (ReK361R), and a C-terminal GFP-tagged version (ReCre1-GFP and ReK361R-GFP) on glucose and malt extract plates.
(TIF)
